# Supplementary material for: A biomimetic chiral-driven ionic gate constructed by pillar[6]arene-based host–guest systems
Source: Nat Commun. 2018 Jul 5;9:2617. doi: 10.1038/s41467-018-05103-w (PMC6033921; doi:10.1038/s41467-018-05103-w)
Supplement: Supplementary file 1 — Supplementary Information [file 41467_2018_5103_MOESM1_ESM.pdf]

Supplementary Information

A biomimetic chiral-driven ionic gate constructed by pillar[6]arene-based host–guest systems

Yue Sun, Fan Zhang, Jiaxin Quan, Fei Zhu, Wei Hong, Junkai Ma, Huan Pang, Yao Sun, Demei Tian, Haibing Li*

Supplementary Figures

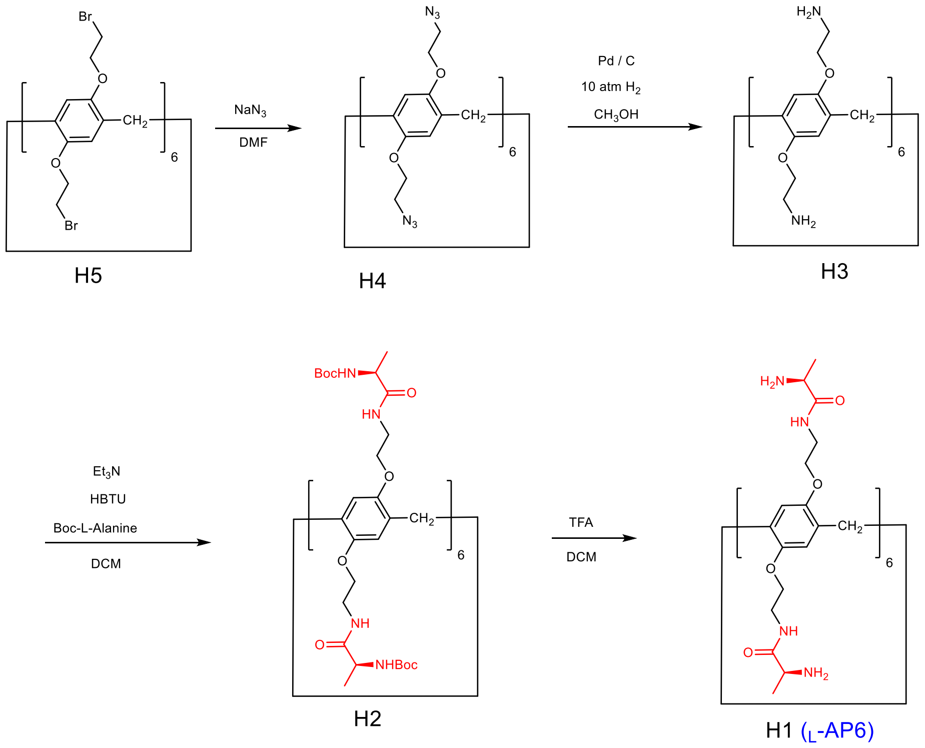

Supplementary Figure 1. The synthesis route to chiral host L-AP6

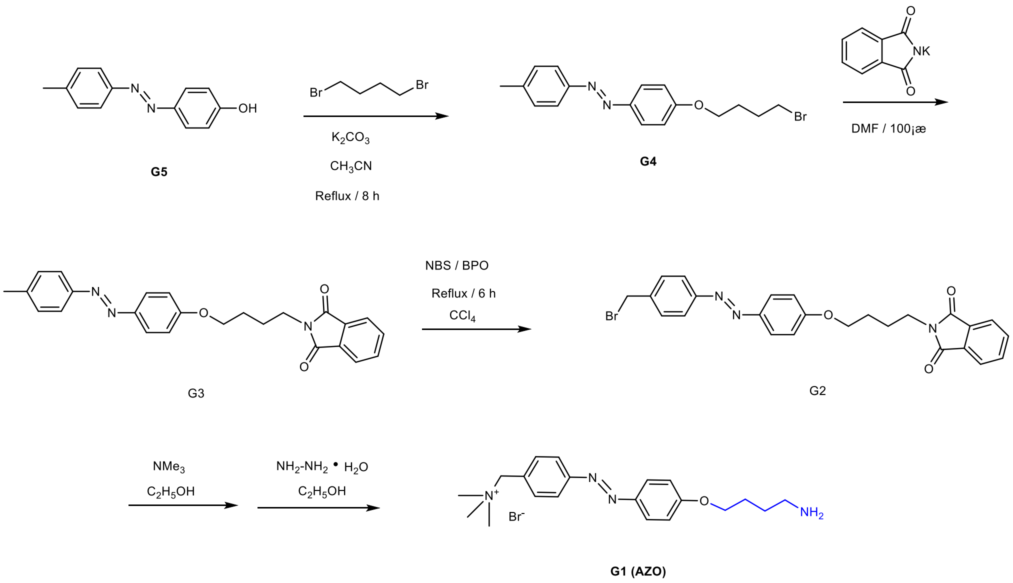

Supplementary Figure 2. The synthesis route for AZO (G1)

<u>^{1}H-NMR titration between $_{L}$-AP6 and AZO</u>

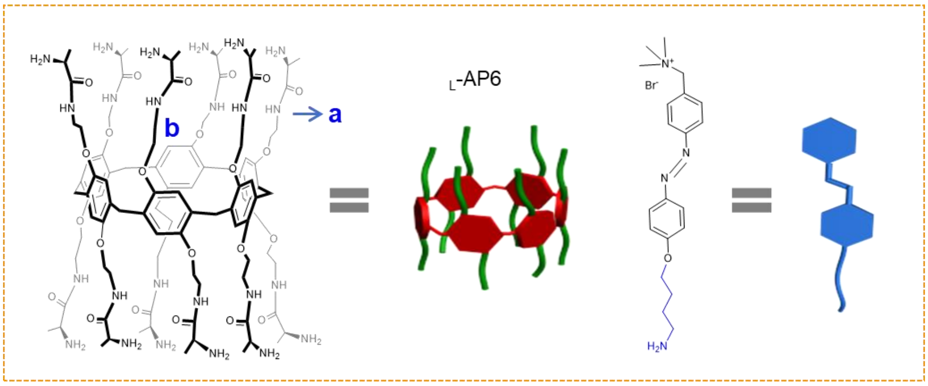

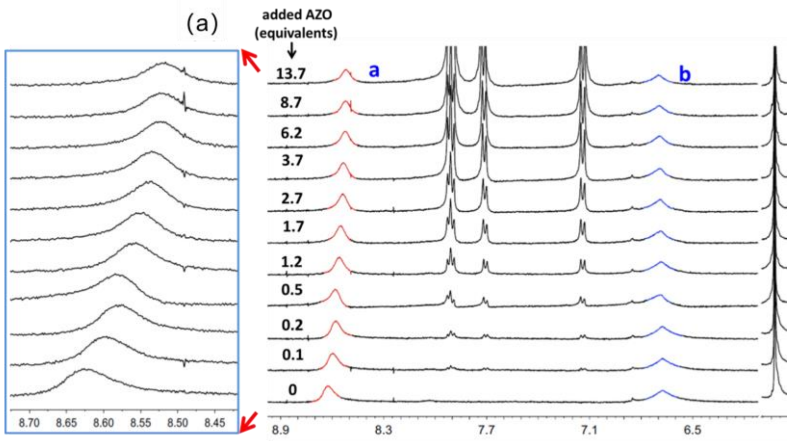

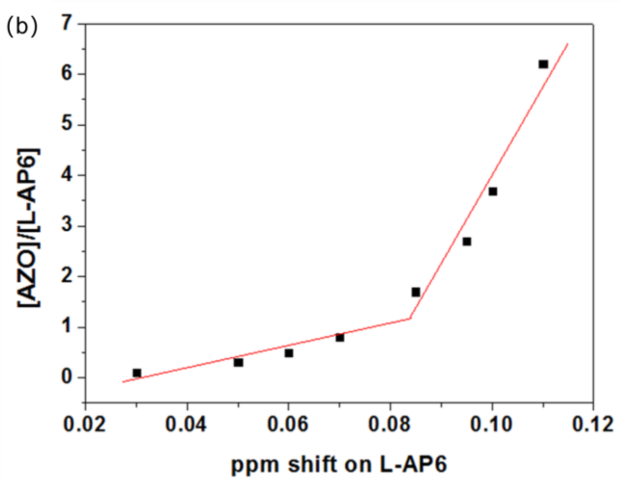

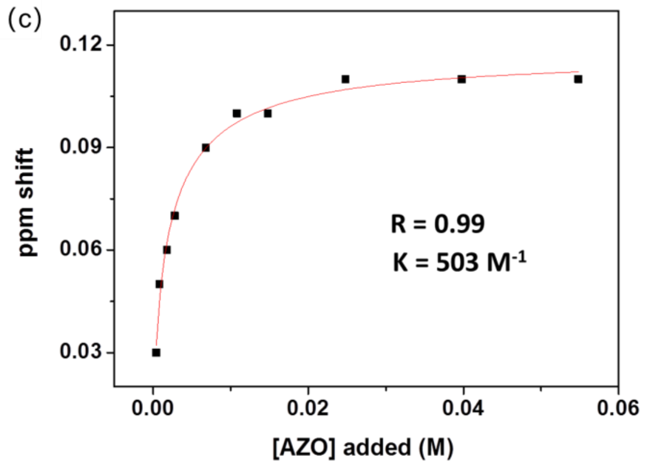

Supplementary Figure 3. **(a)** ^{1}H NMR (400 MHz) binding study of complex L-AP6 vs AZO in DMSO; **(b)** The mole ratio plot for the complexation between L-AP6 and AZO, indicating a 1:1 stoichiometry; **(c)** The non-linear curve-fitting (NMR titrations) for the complexation of L-AP6 (4.0 mM) with different concentration of AZO. The association constant (K_a) of L-AP6 and AZO was calculated to be 503 M^{-1}

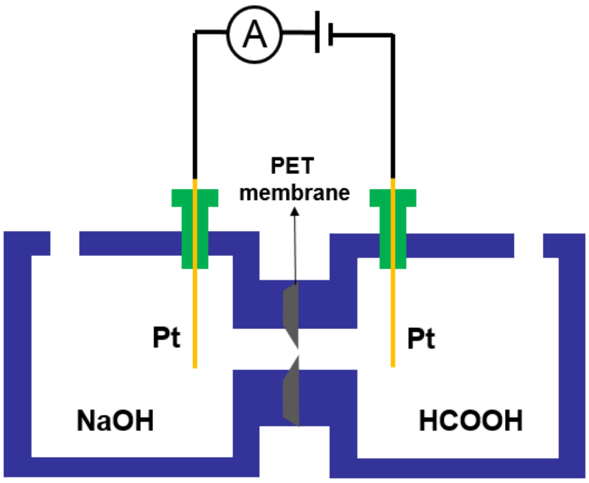

Supplementary Figure 4. Schematic image for etching conical nanochannel in a conductivity cell

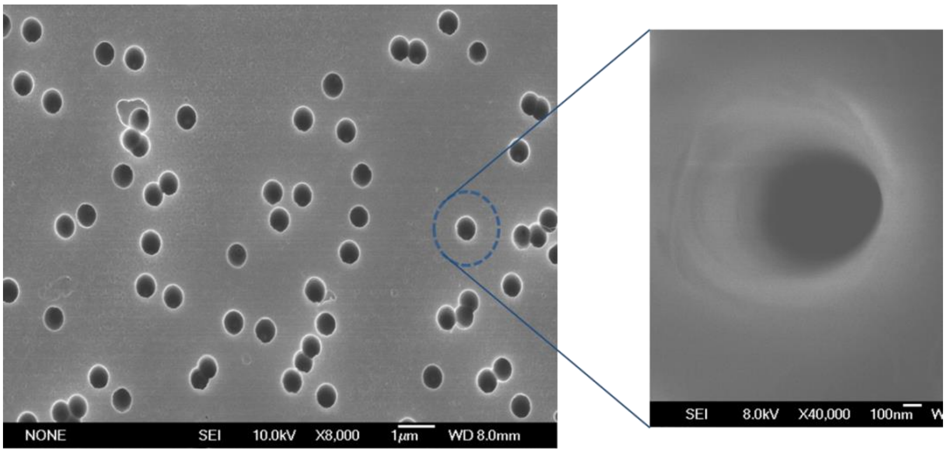

Supplementary Figure 5. SEM image of the base side and the tip side of the conical nanochannel in PET porous membrane channels (10^7channels cm^{-2}). It shows that the diameter of the large pening (base) of the conical nanochannel was approximately 580 nm, while that of the narrow opening (tip) at the opposite face was approximately 18 nm

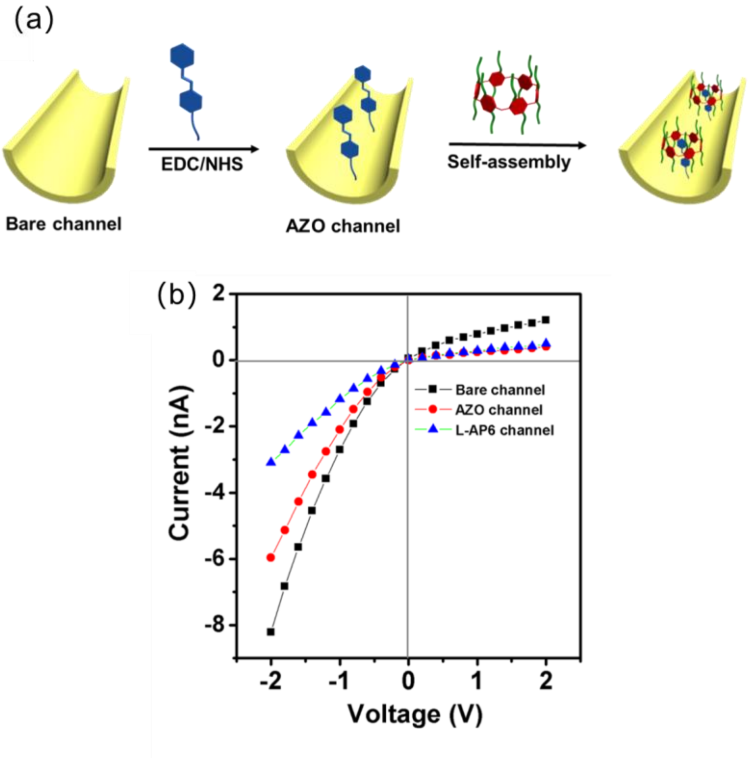

Supplementary Figure 6. **(a)** Schematic description of modification process in nanochannel; **(b)** *I*–*V* characteristics of L-AP6-assembled nanochannel

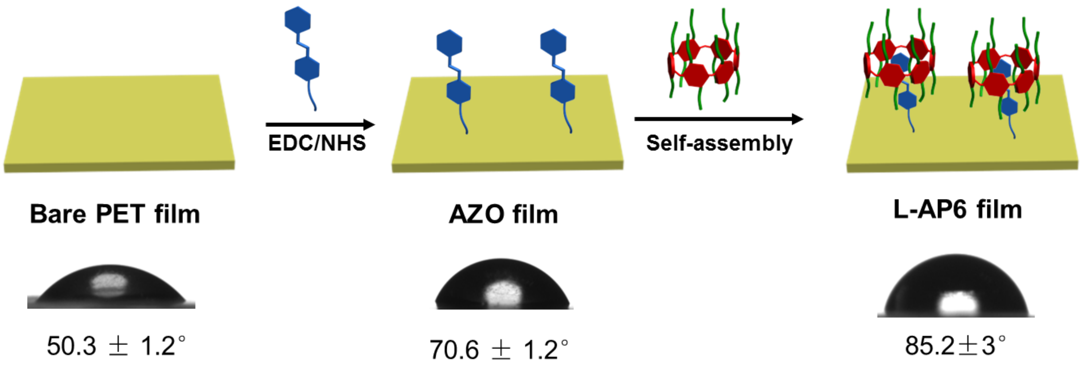

Supplementary Figure 7. The wettability change of L-AP6-assembled nanochannel

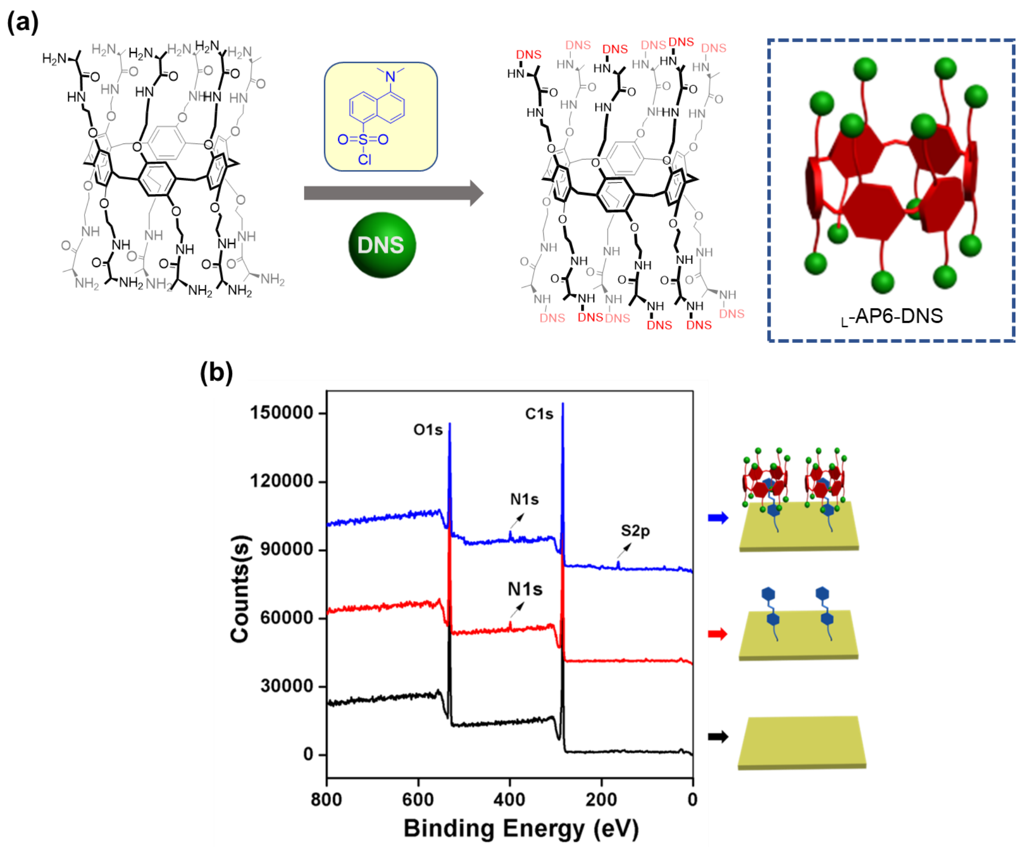

Supplementary Figure 8. XPS spectra of PET films before and after modification. **(a)** L-AP6 was labeled with dansyl chloride; **(b)** The control was referenced to the bare film (black). The modified AZO was referenced to the film after the modification of AZO (red), and the modified L-AP6-DNS was referenced to the film after the modification of L-AP6-DNS (blue). The results indicates that chiral L-AP6 was modified on the surface of the film successfully

Supplementary Table 1. The XPS data of the PET film before modification

Name	Start BE	Peak BE	End BE	Height (CPS)	FWHM (eV)	Area (P) CPS.eV	Atomic %
C1s	292.57	284.78	280.57	18855.95	1.28	33261.59	78.69
O1s	538.17	531.88	526.77	11284.32	2.1	25364.23	19.47

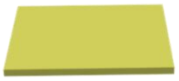

Bare PET films

Supplementary Table 2. The XPS data of the PET film after AZO modification

Name	Start BE	Peak BE	End BE	Height (CPS)	FWHM (eV)	Area (P) CPS.eV	Atomic %
C1s	291.24	284.63	281.6	67547.18	1.93	163723.8	79.19
O1s	536.51	531.8	528.25	34593.4	2.64	97589.71	16.48
N1s	403.8	399.39	396.25	5836.22	2.01	1444.38	2.36

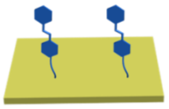

Modified AZO

Supplementary Table 3. The XPS data of the AZO-modified PET film assembled with L-AP6-DNS

Name	Start BE	Peak BE	End BE	Height (CPS)	FWHM (eV)	Area (P) CPS.eV	Atomic %
C1s	293.61	285.33	281.3	23387.85	1.25	44473.38	79.06
O1s	538.12	532.76	527.16	14081.68	1.82	38929.4	18.88
N1s	404.42	402.01	397.68	317.49	1.28	1300.01	1.88
S2p	171.7	168.32	165.05	524.64	2.3	672.53	1.67

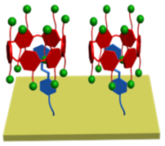

Assemble L-AP6-DNS

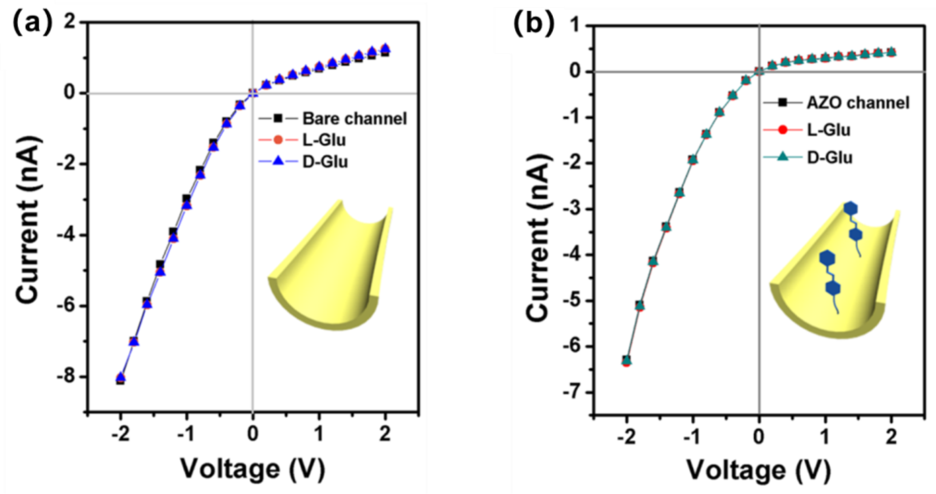

Supplementary Figure 9. The *I-V* curve of the **(a)** bare nanochannels and **(b)** AZO modified nanochannels in the electrolyte adding 10^{-3} M L-Glu and D-Glu, respectively. No obvious current change indicated the bare nanochannel and Azo-modified nanochannels was not selective for Glu

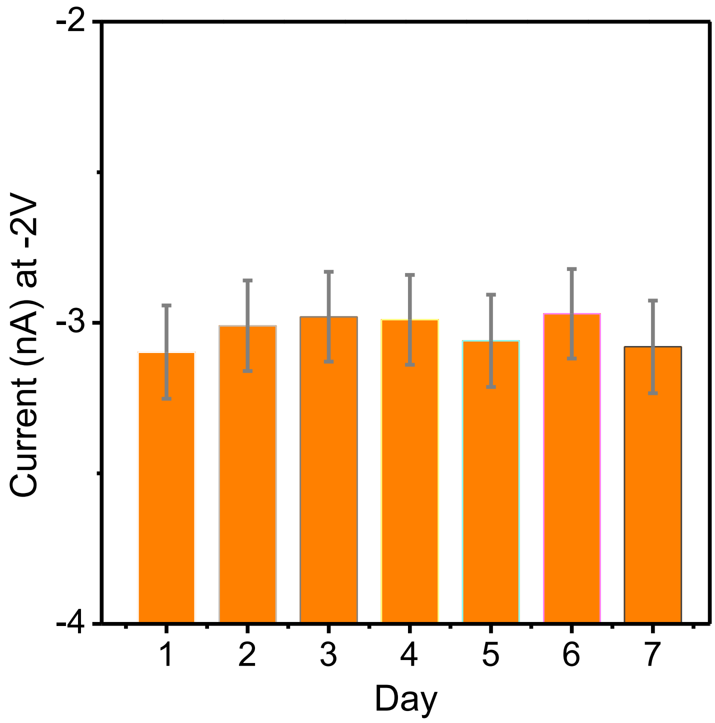

Spplementary Figure 10. The stability of the complex forming by the L-AP6 and AZO in the nanochannel. Standard deviation is ± 5% and are used for describing the error bars. Each data in two cases are tested five times, respectively

Supplementary Table 4. *K* values (binding constants) for the different saccharides enantiomers calculated using the Langmuir equation

Saccharides	K/M^{-1}	$K_{(D\text{-}Saccharides)} / K_{(L\text{-}Saccharides)}$
D-Glu	$2.63X10^5$	32.3
L-Glu	$8.14X10^3$	
D-Gal	$5.86X10^4$	2.5
L-Gal	$2.37X10^4$	
D-Xyl	$4.91X10^4$	2.2
L-Xyl	$2.25X10^4$	

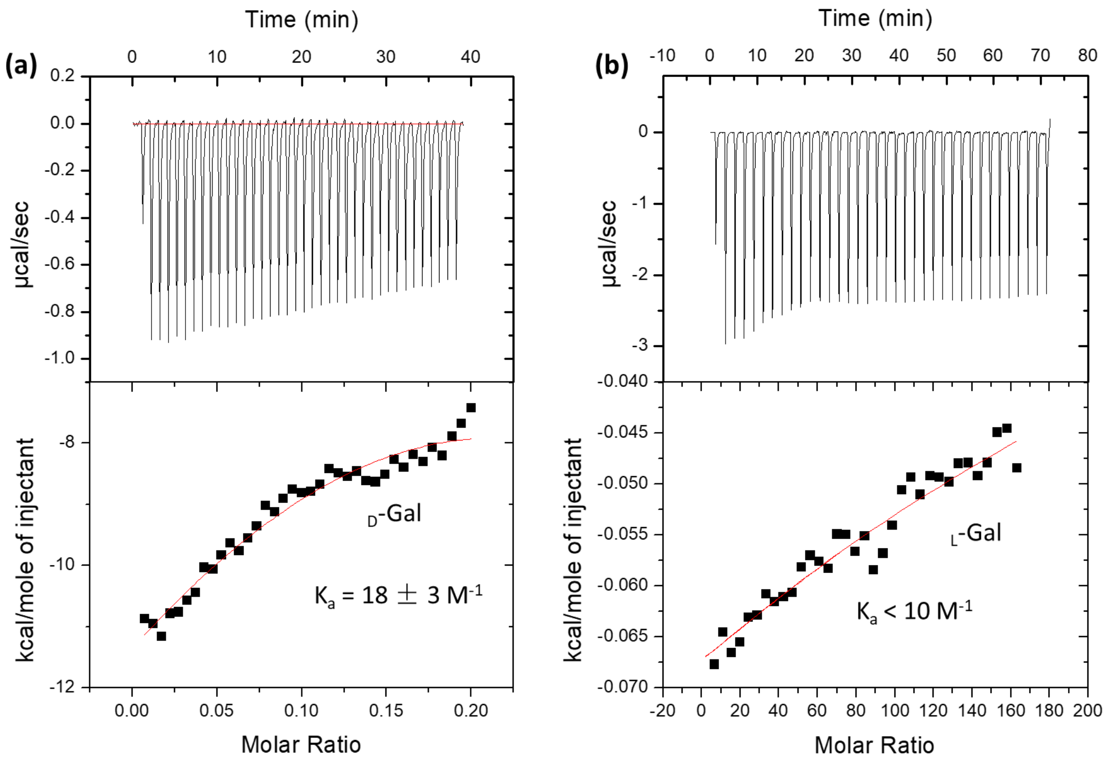

Supplementary Figure 11. The L-AP6 (1.0 mM) with Gal enantiomers (200 mM) in water at 25 °C: **(a)** with D-Gal; **(b)** with L-Gal

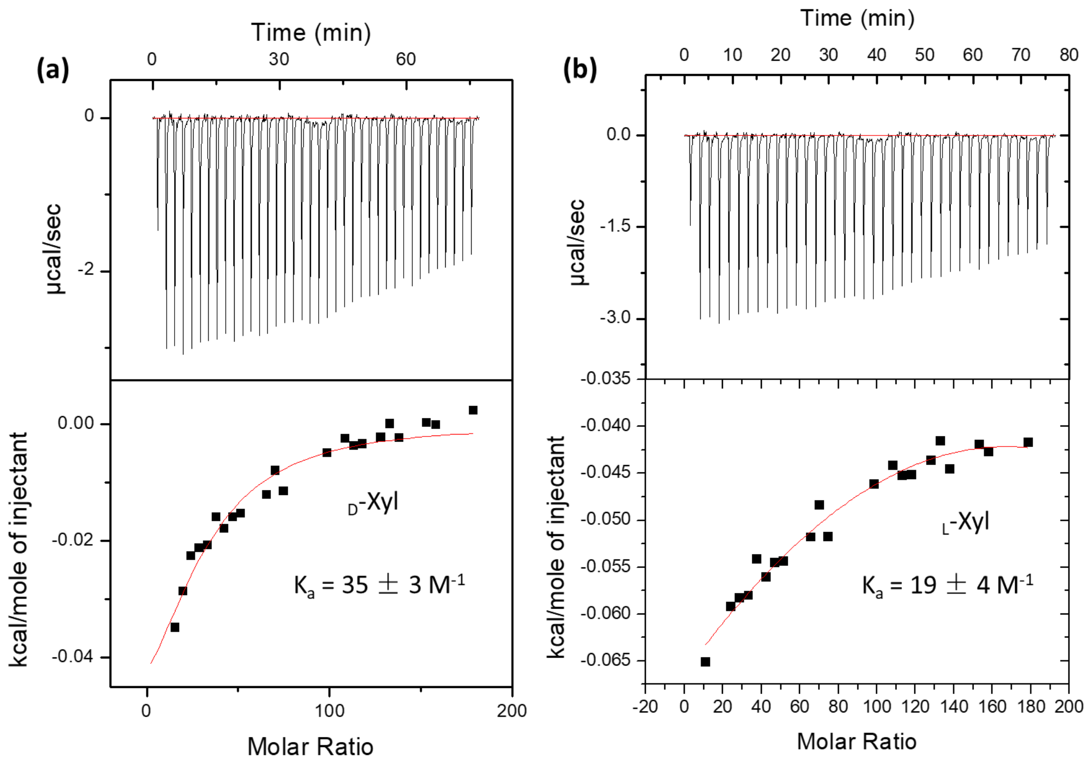

Supplementary Figure 12. The L-AP6 (1.0 mM) with Xyl enantiomers (200 mM) in water at 25 °C: **(a)** with D-Xyl; **(b)** with L-Xyl

^{1}H-NMR titration between L-Glu and complex L-AP6-AZO

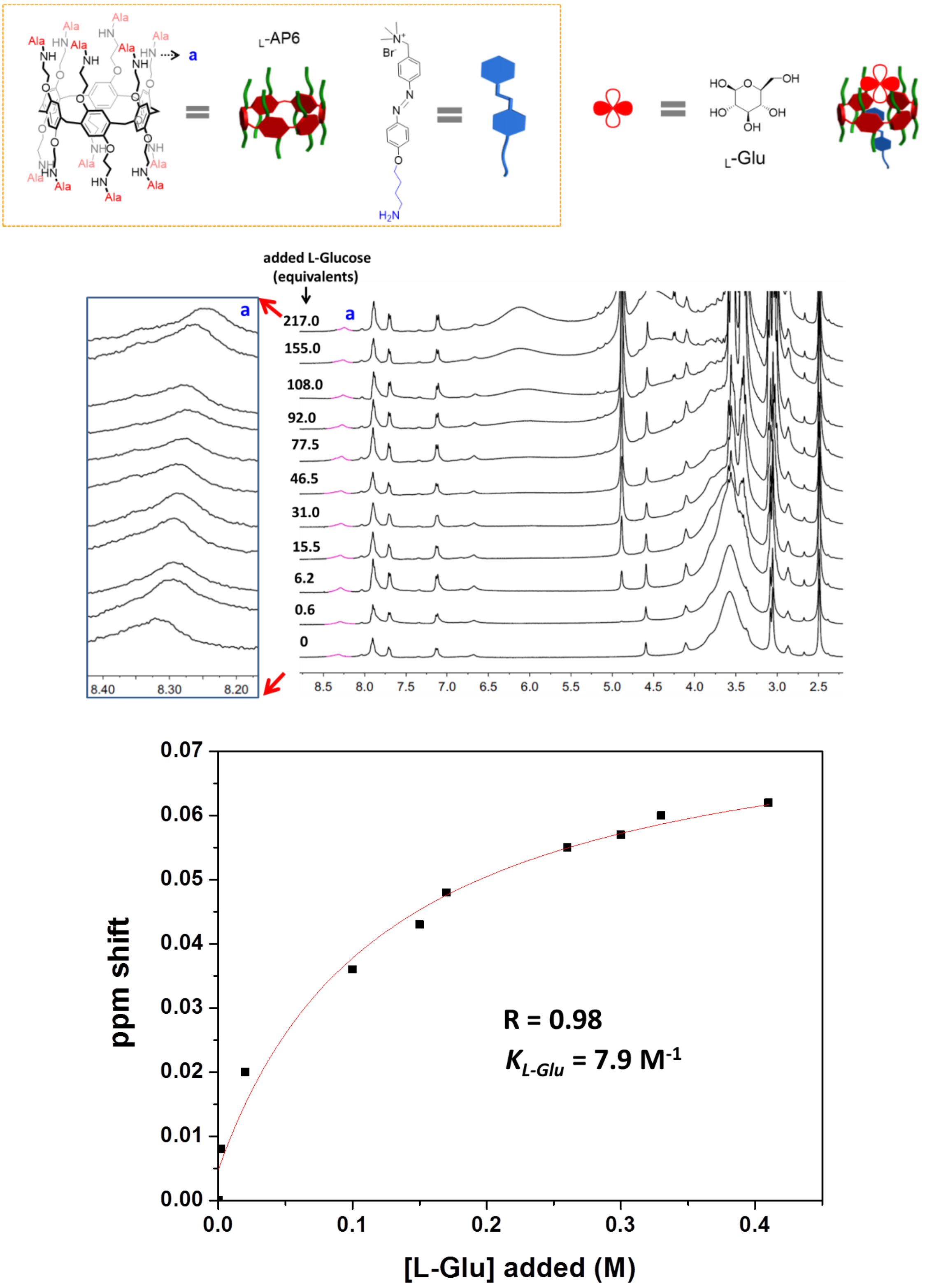

Supplementary Figure 13. Experimental values for the ^{1}H NMR (400 MHz) binding study of complex (L-AP6-AZO) *vs* L-Glu in DMSO. limiting Δδ = -0.0621 ppm

^{1}H-NMR titration between D-Glu and complex L-AP6-AZO

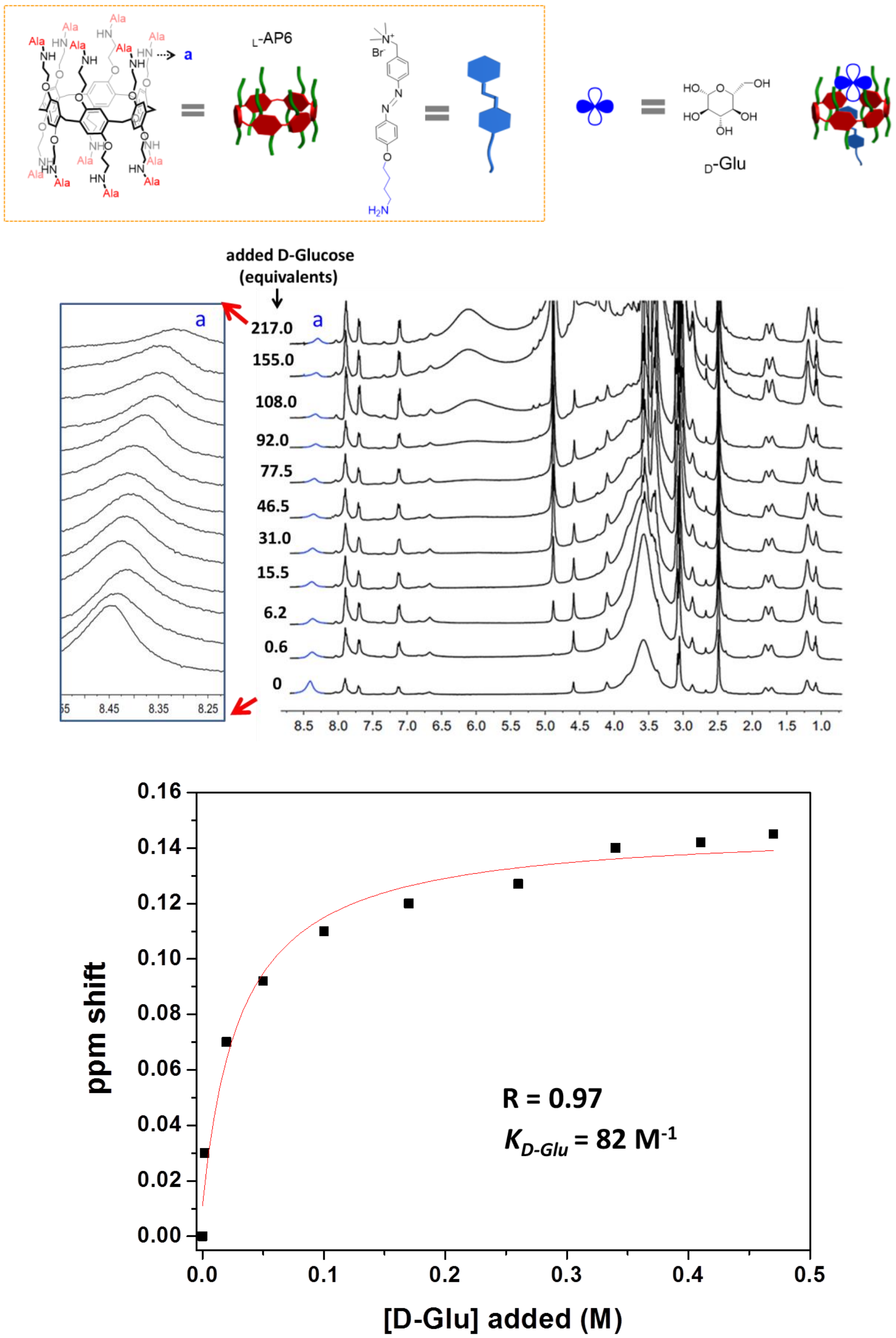

Supplementary Figure 14. Experimental values for the ^{1}H NMR (400 MHz) binding study of complex (L-AP6-AZO) *vs* D-Glu in DMSO. limiting Δδ = -0.147 ppm

Gaussion Calculation between D-Glu/L-Glu and complex (L-AP6-AZO)

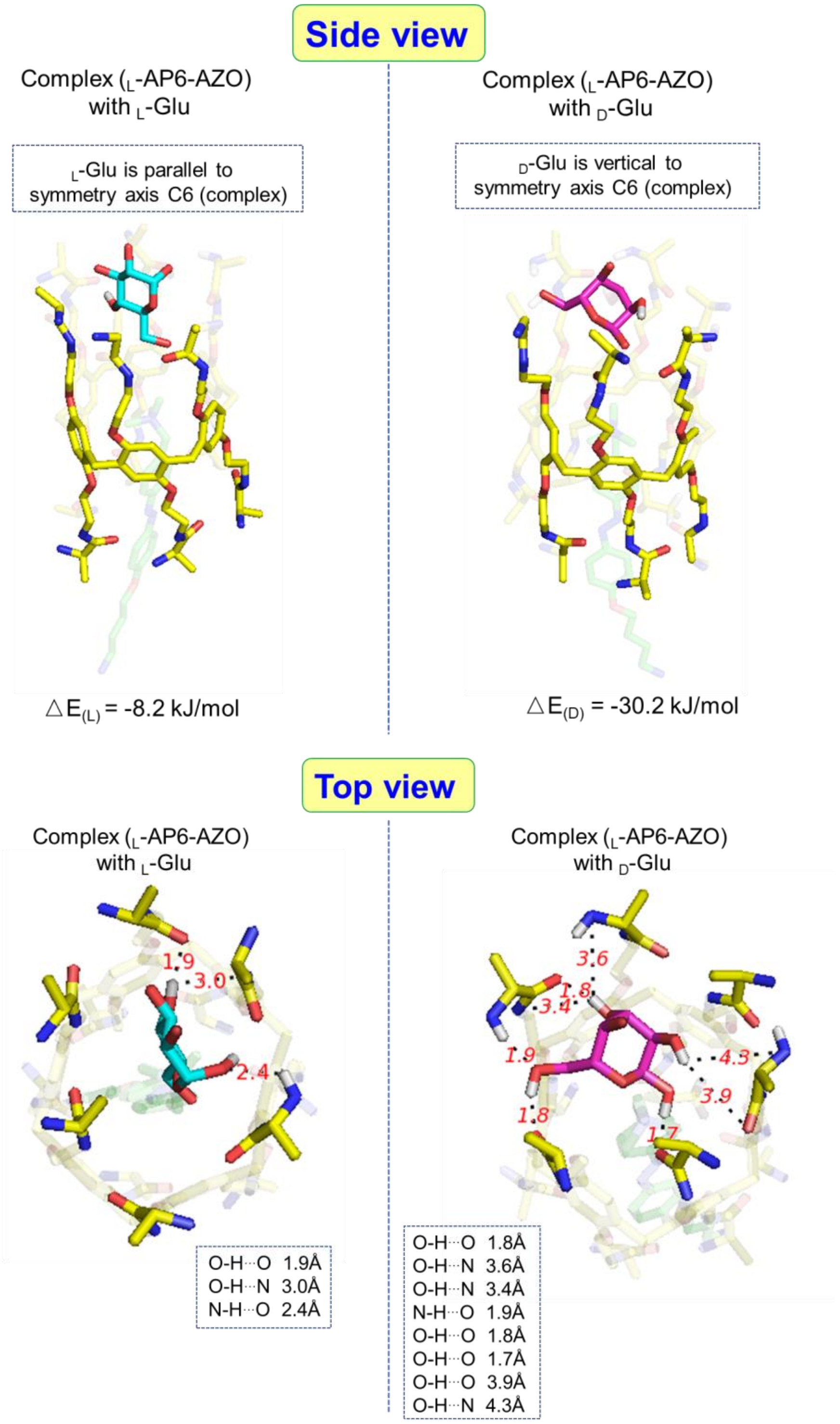

Supplementary Figure 15. Energy-minimized complex of L-AP6 with L-Glu (left) or D-Glu (right), optimized at the B3LYP/6–31G* level. This result show that complex (L-AP6-AZO) prefer to bind D-Glu

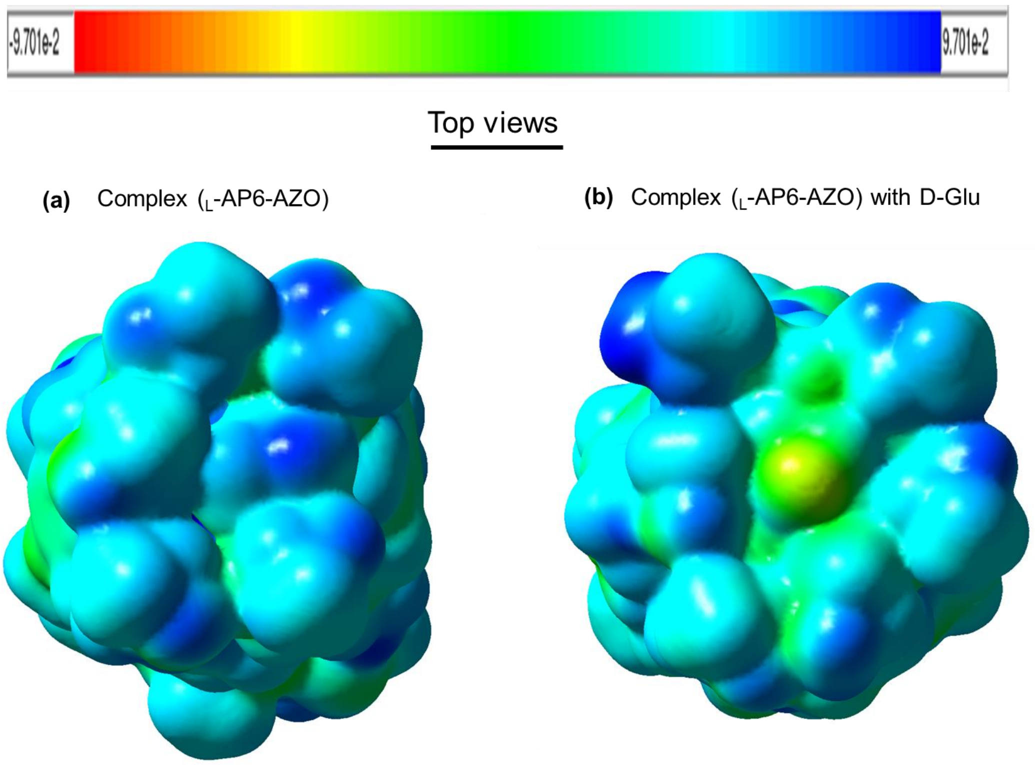

Supplementary Figure 16. Electron density of **(a)** L-AP6-AZO and **(b)** L-AP6-AZO binding D-Glu (right), optimized at the B3LYP/6–31G* level

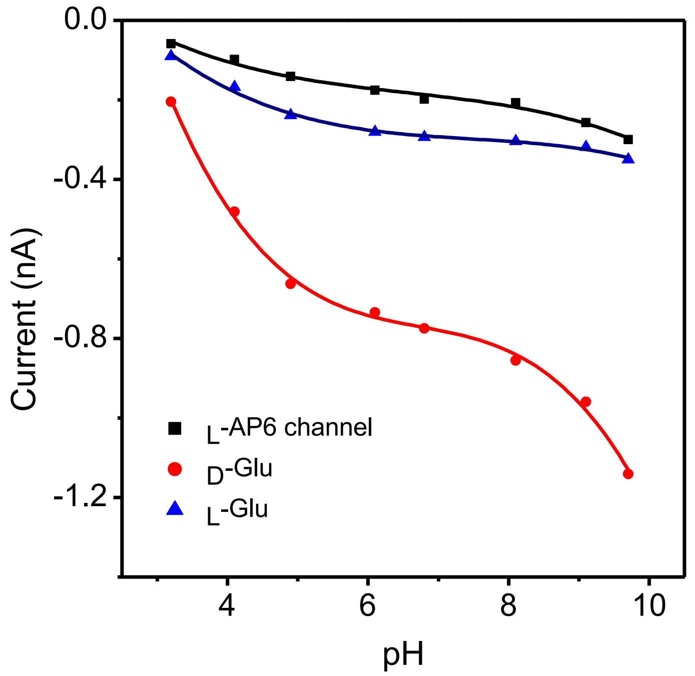

Supplementary Figure 17. Relationship between the current at -2 V and the pH value of the solution

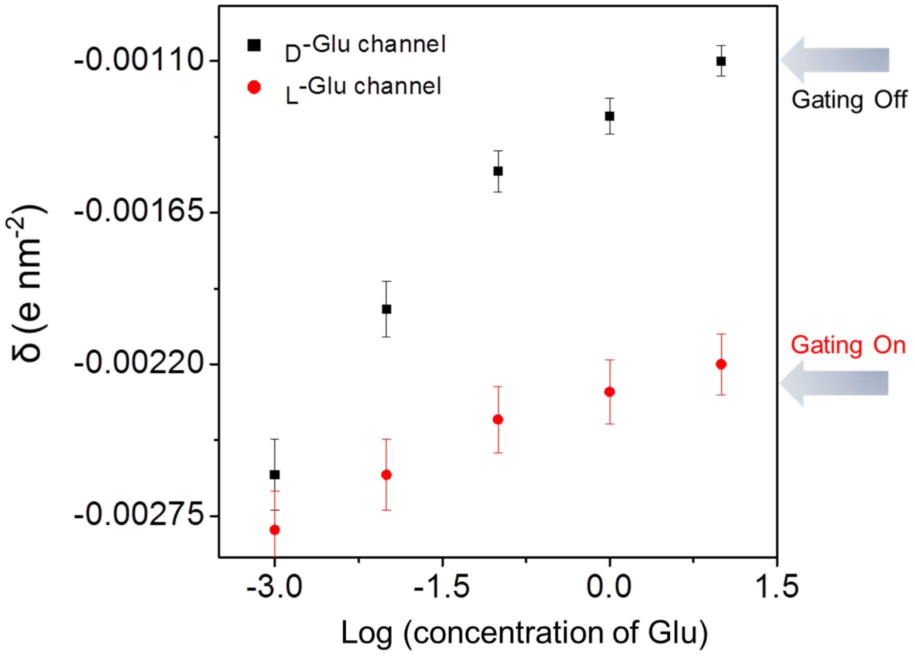

Supplementary Figure 18. Surface charge density *vs* Log (concentration of Glu). It indicates that the surface charge density decreased gradually with the increasing of concentration. Standard deviation is ± 5% and are used for describing the error bars. Each data in two cases are tested five times, respectively

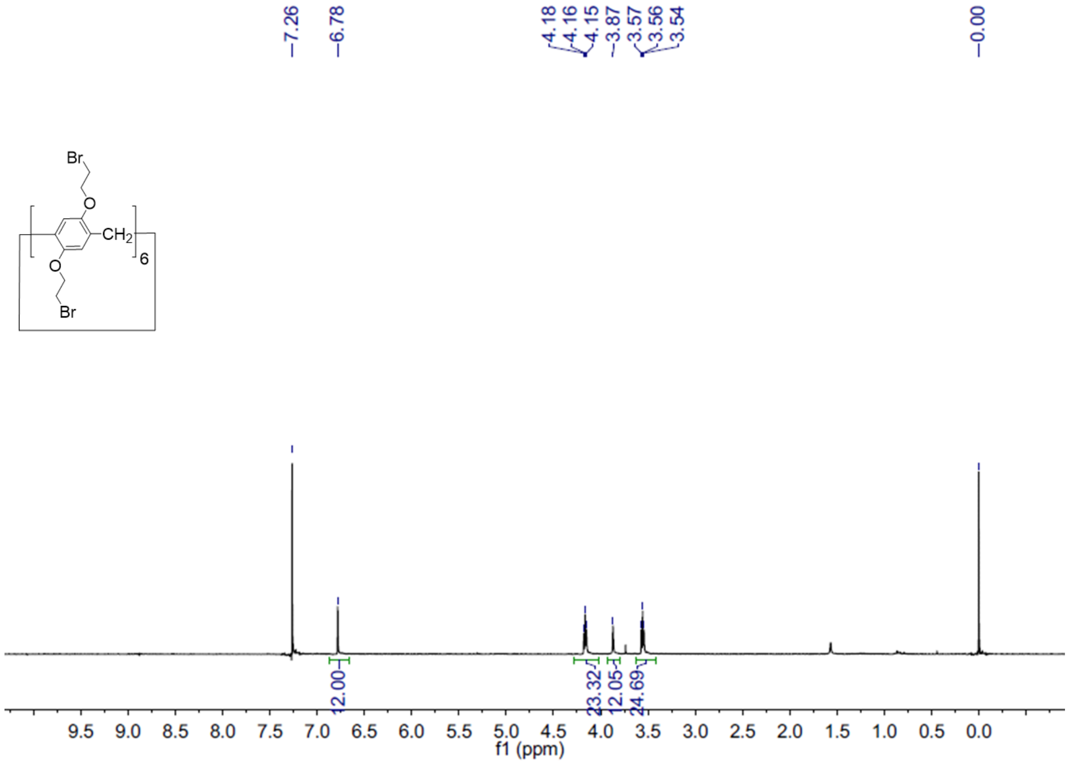

Supplementary Figure 19. ^{1}H NMR spectrum (400 MHz) of compound H5 in $CDCl_3$

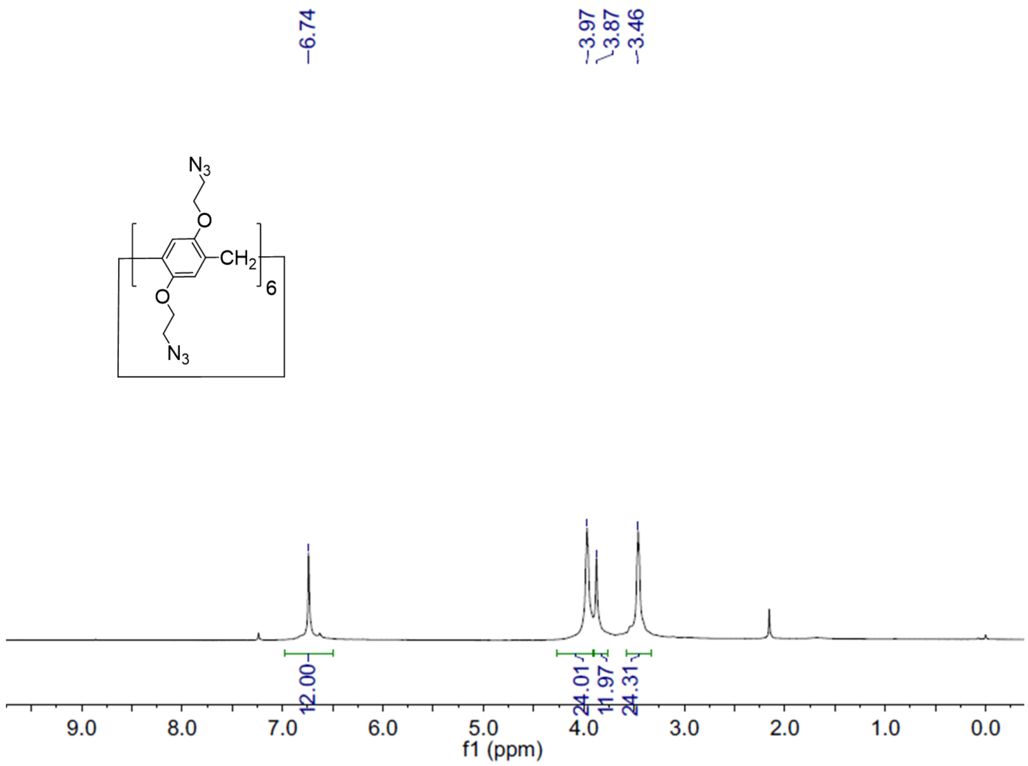

Supplementary Figure 20. ^{1}H NMR spectrum (400 MHz) of compound H4 in $CDCl_3$

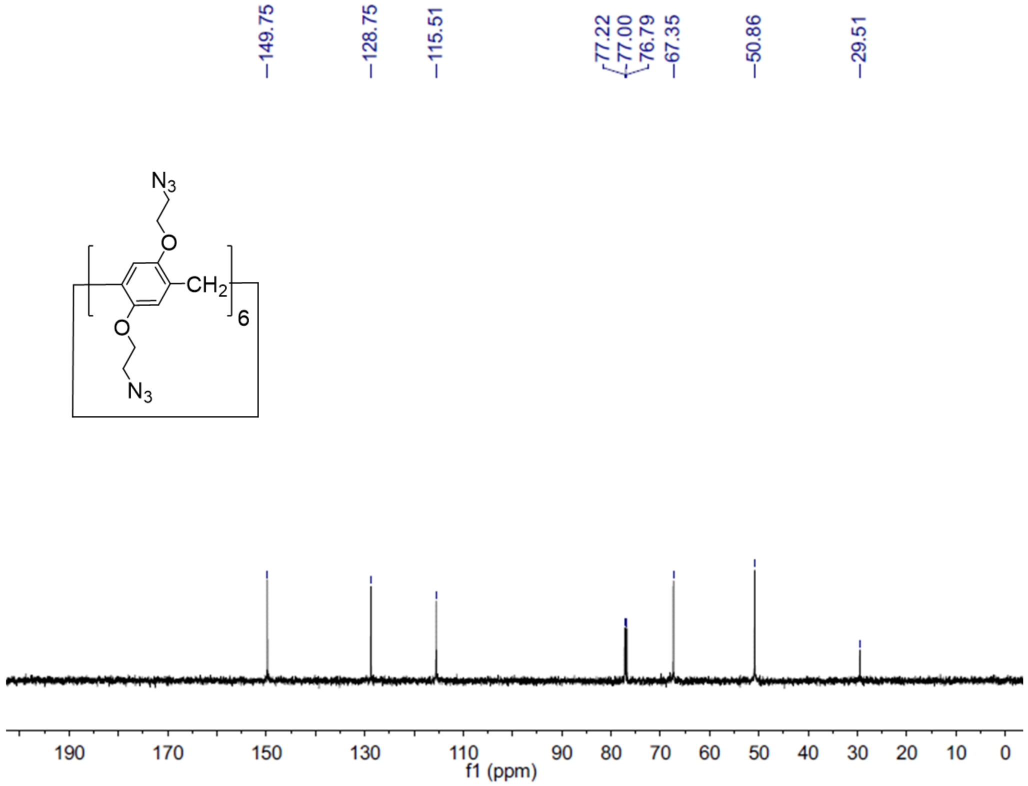

Supplementary Figure 21. ^{13}C NMR spectrum (100 MHz) of compound H4 in $CDCl_3$

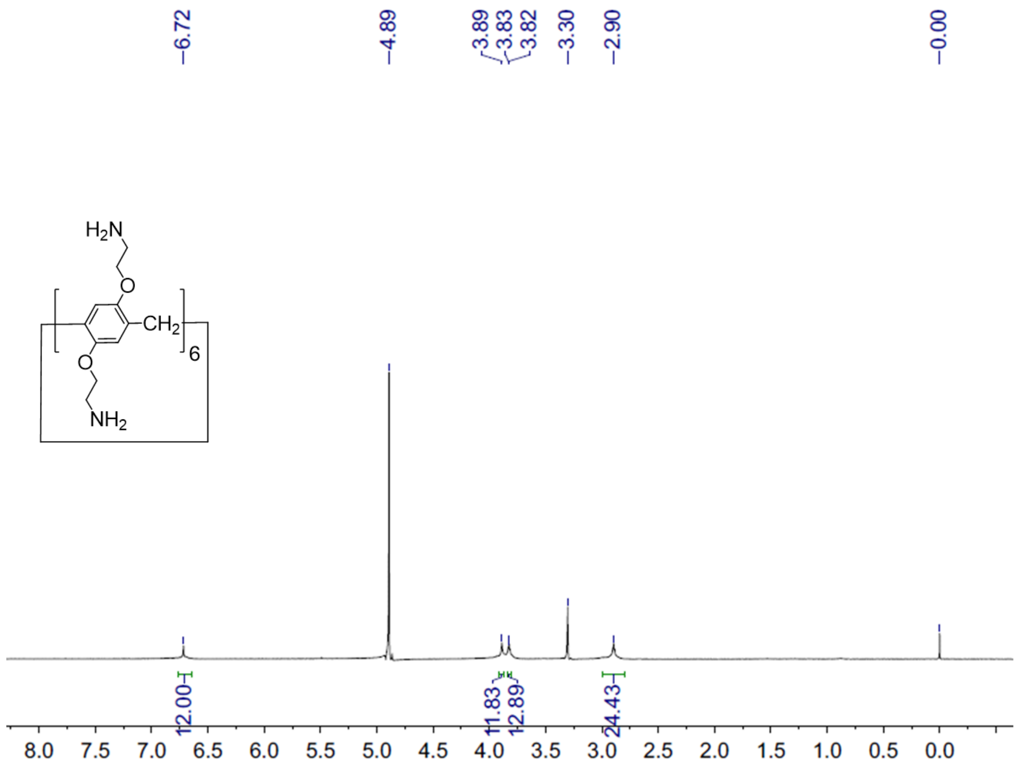

Supplementary Figure 22. ^{1}H NMR spectrum (400 MHz) of compound H3 in CH_3OH-d_4

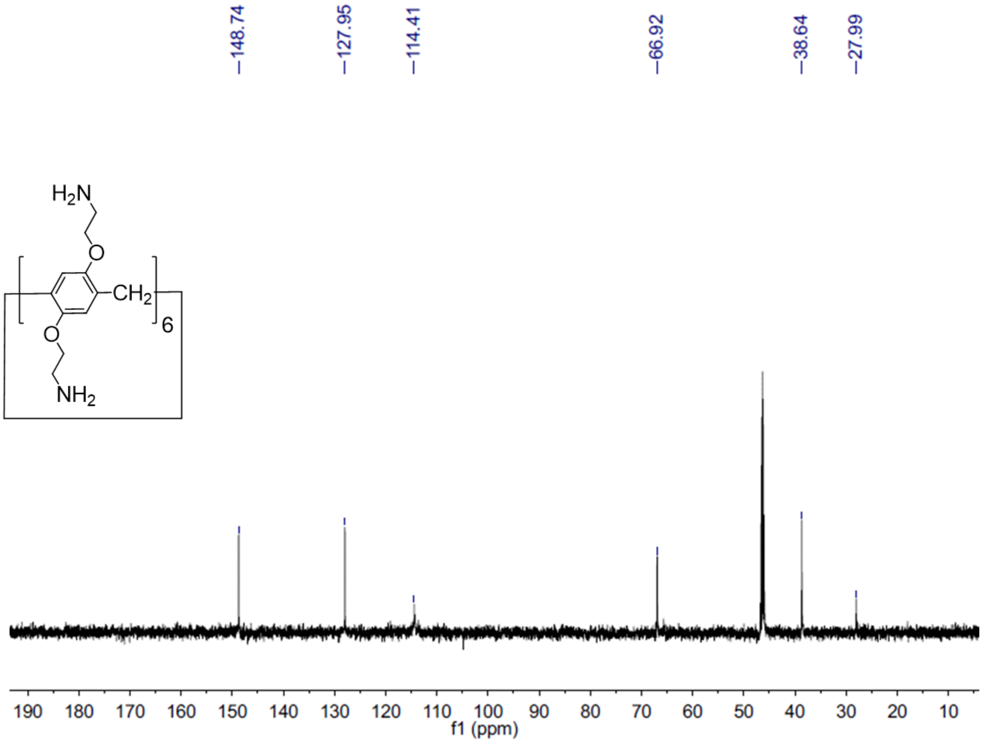

Supplementary Figure 23. ^{13}C NMR spectrum (100 MHz) of compound **H3** in CH_3OH-d_4

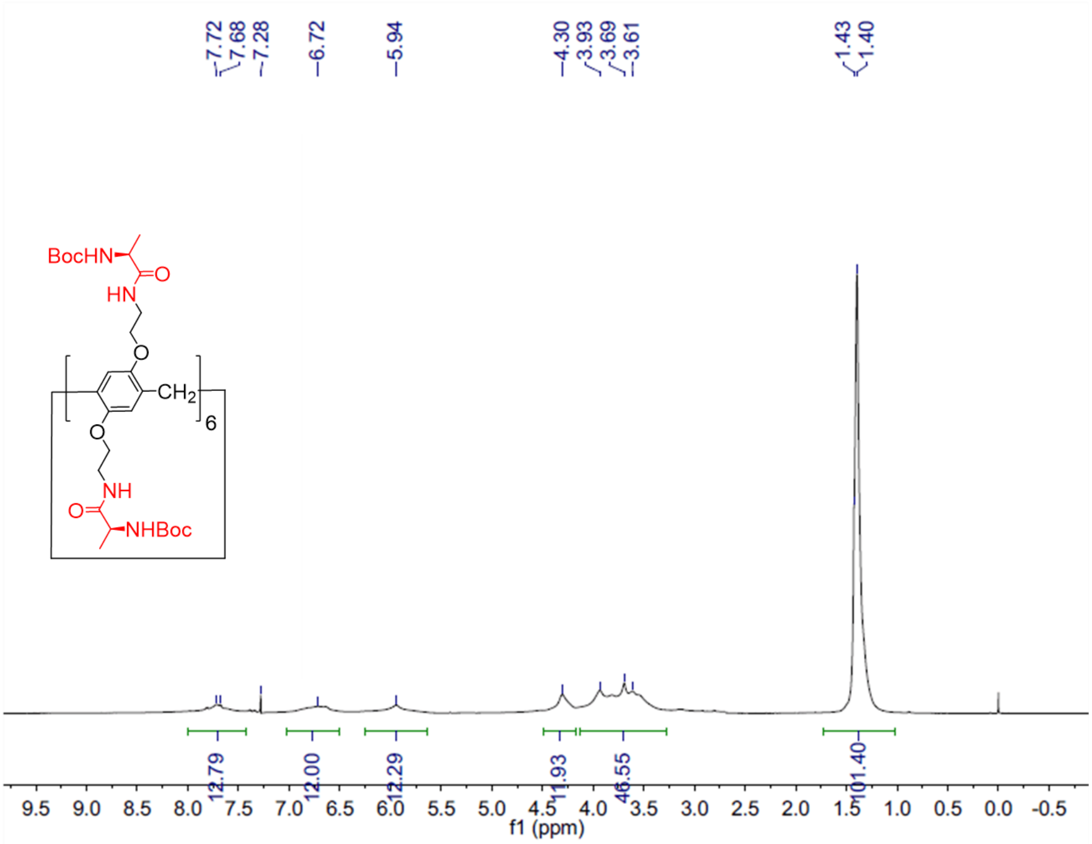

Supplementary Figure 24. ^{1}H NMR spectrum (400 MHz) compound **H2** in $CDCl_3$

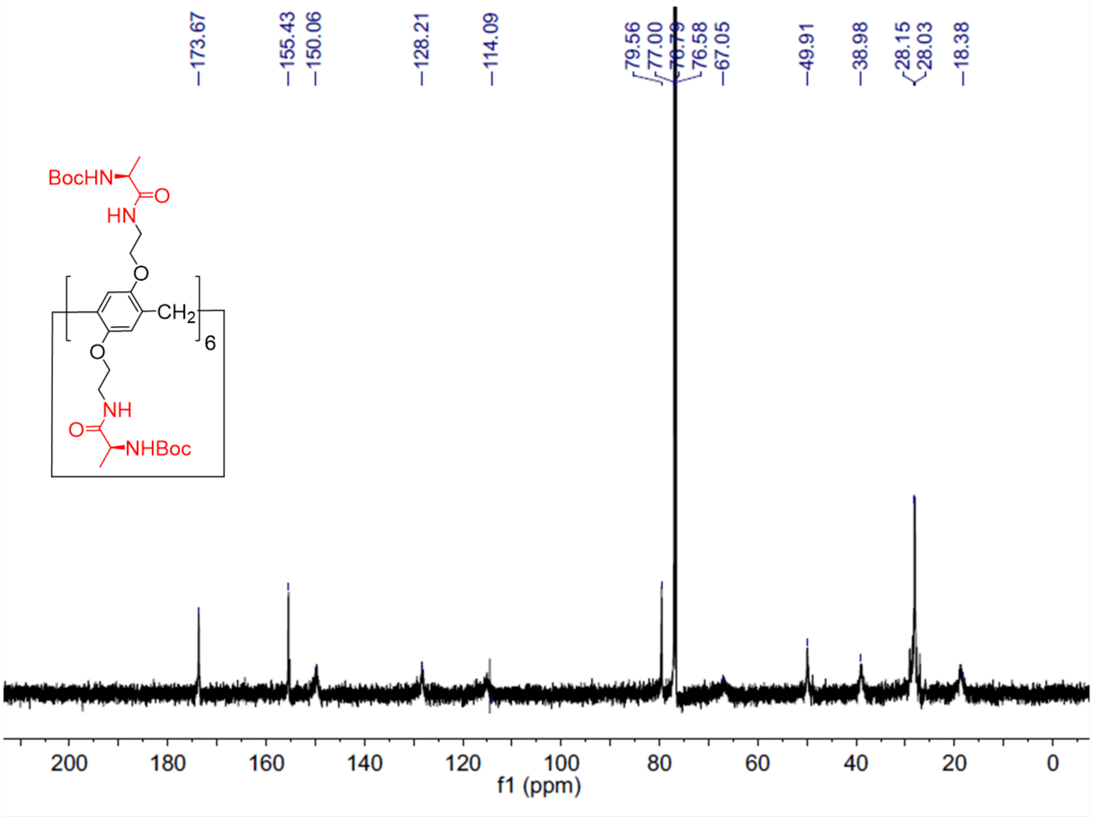

Supplementary Figure 25. ^{13}C NMR spectrum (100 MHz) compound **H2** in $CDCl_3$

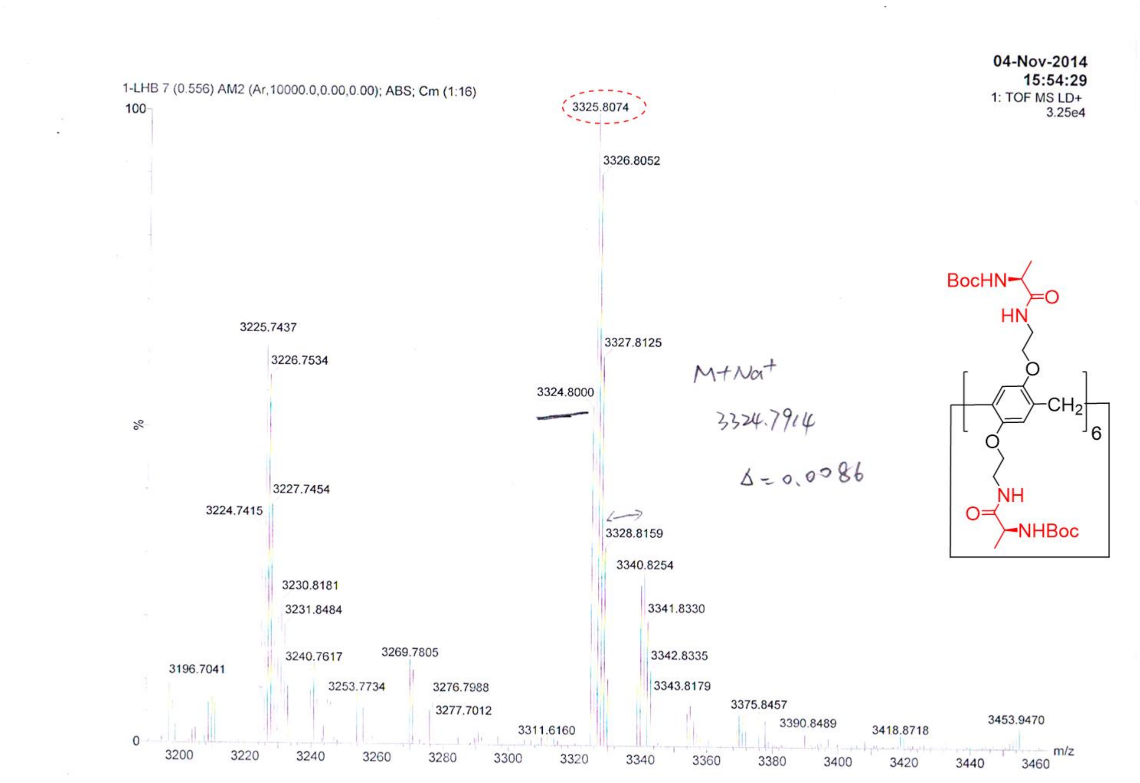

Supplementary Figure 26. Mass spectrum of compound **H2**

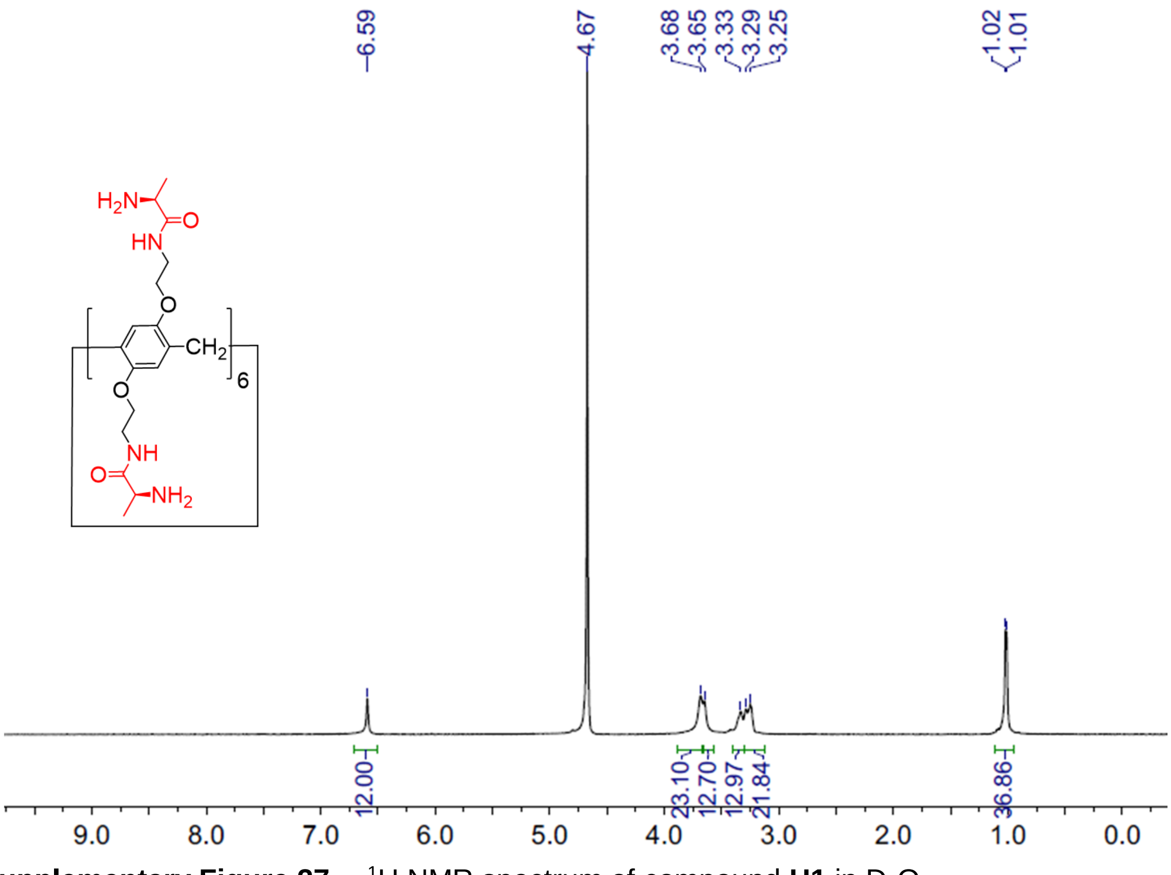

Supplementary Figure 27. ^{1}H NMR spectrum of compound **H1** in D_2O

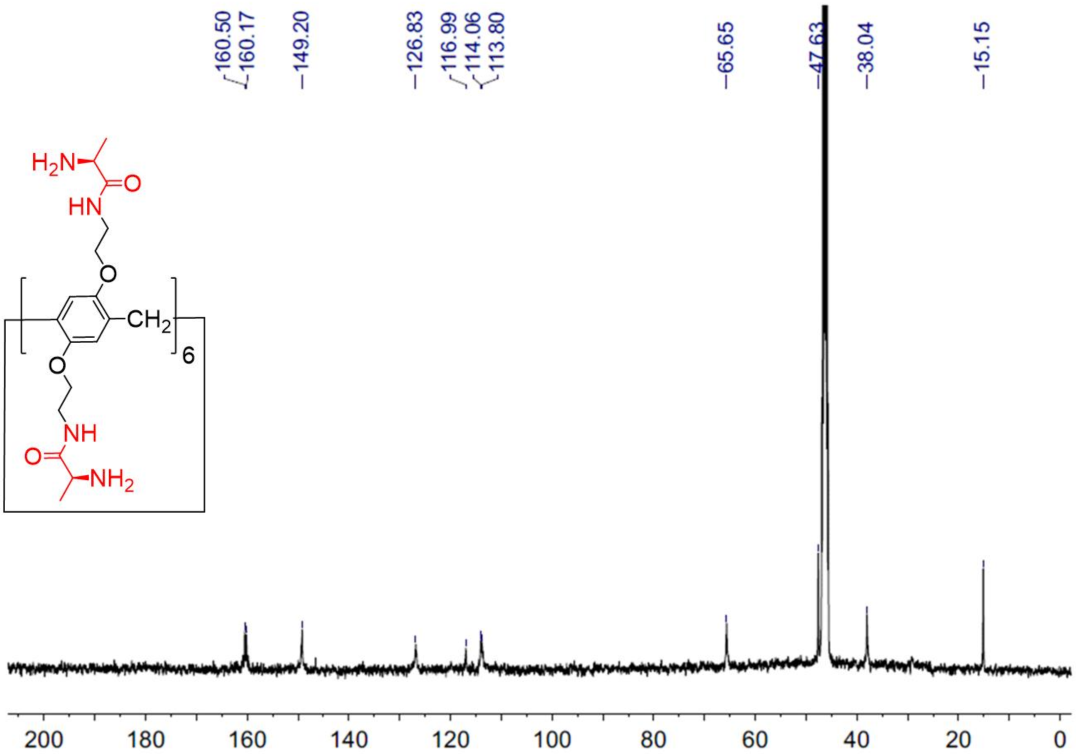

Supplementary Figure 28. ^{13}C NMR spectrum (100 MHz) compound H1 in CH_3OH-d_4

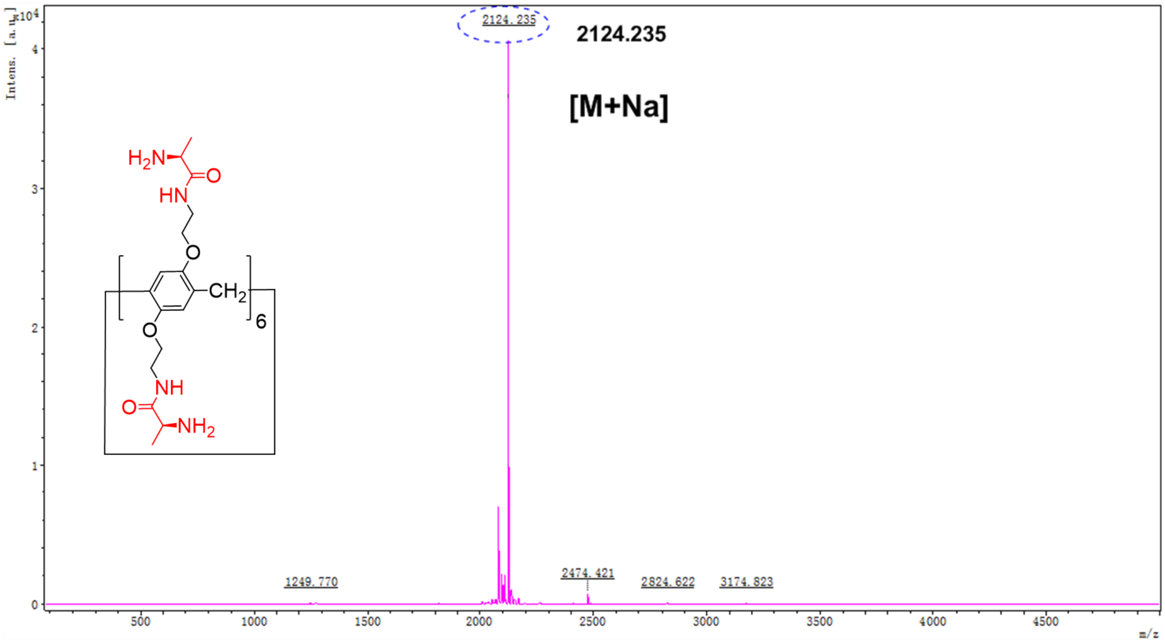

Supplementary Figure 29. Mass spectrum of compound **H1**

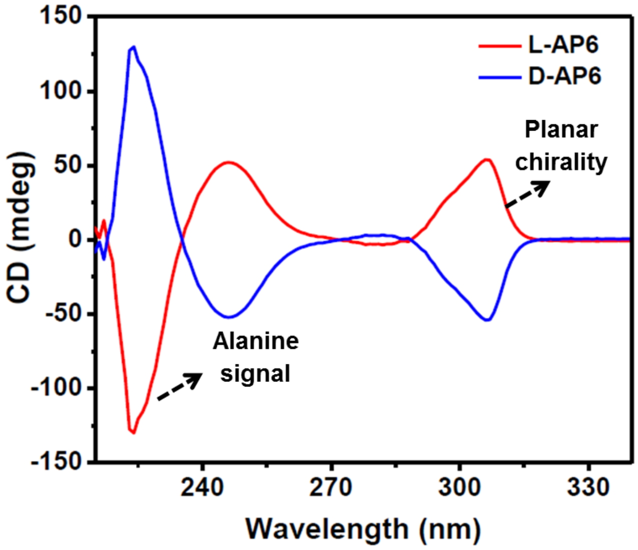

Supplementary Figure 30. CD spectram of chiral compound **H1**

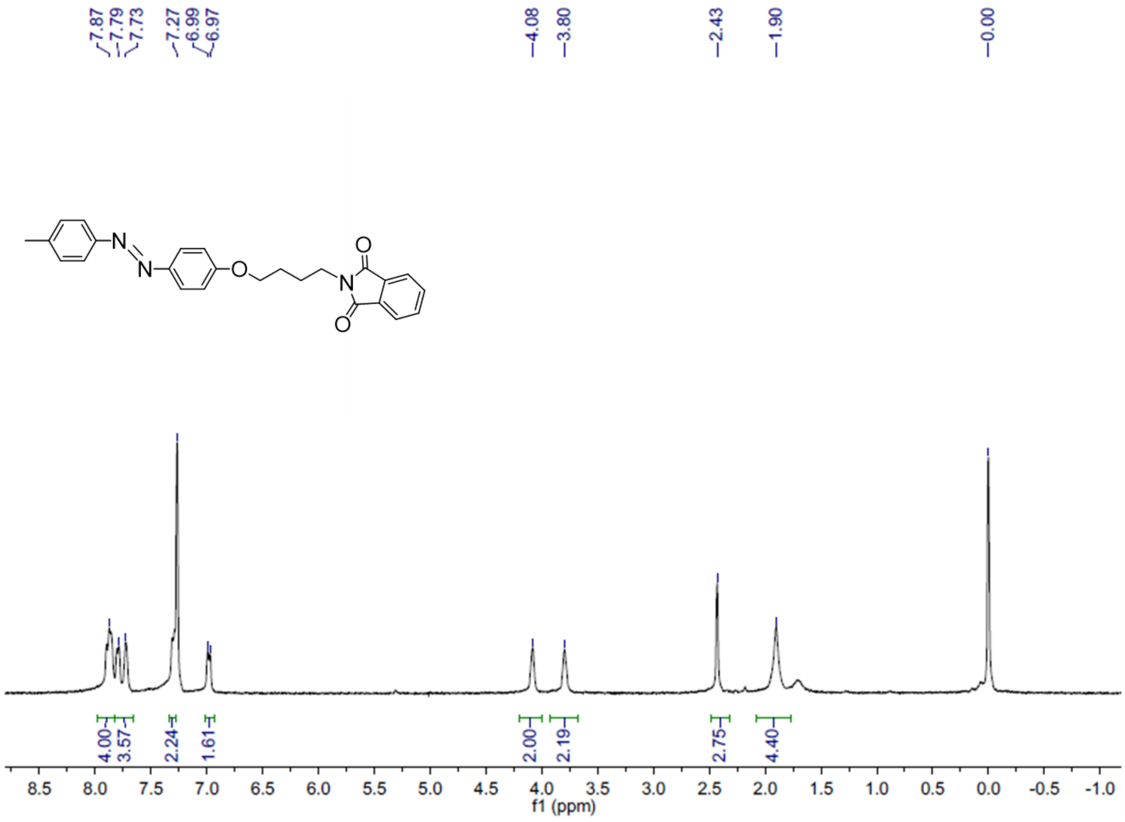

Supplementary Figure 31. ^{1}H NMR spectrum (400 MHz) of compound **G3** in $CDCl_3$

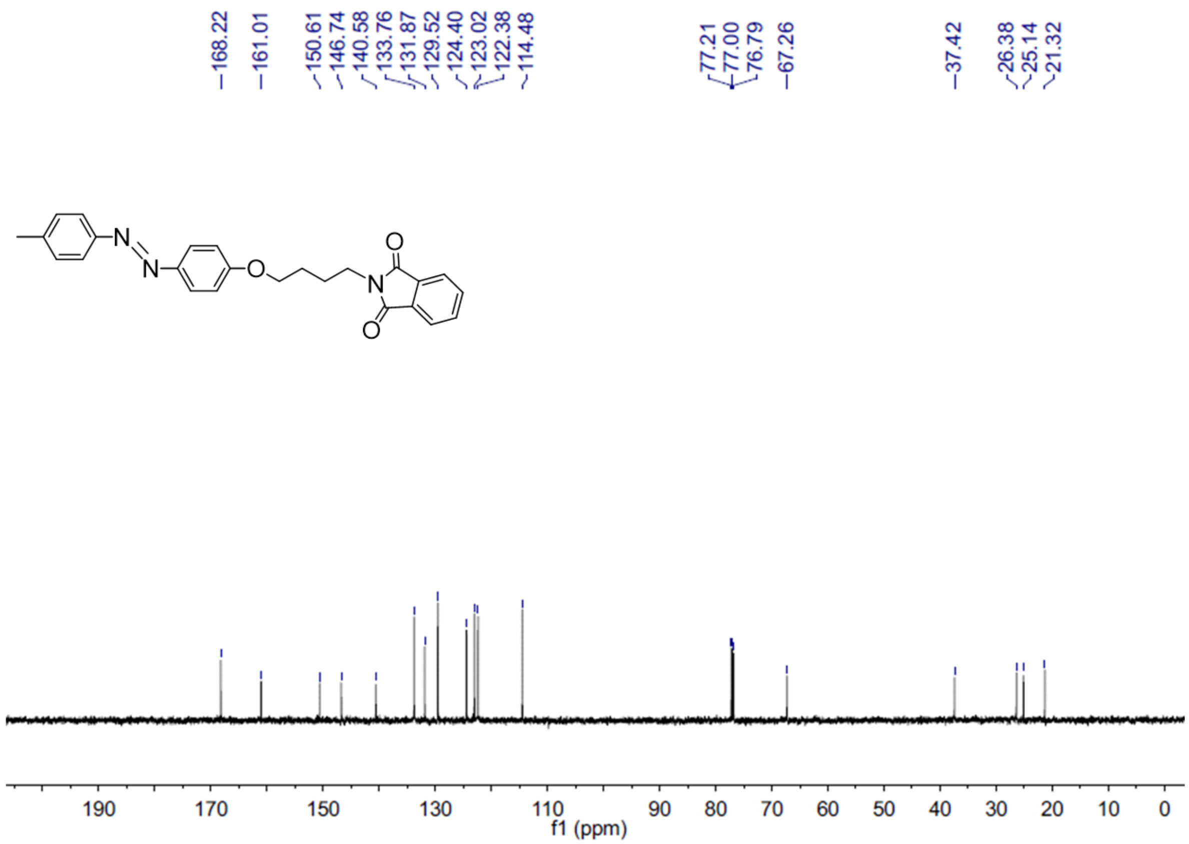

Supplementary Figure 32. ^{13}C NMR spectrum (100 MHz) of **G3** in $CDCl_3$

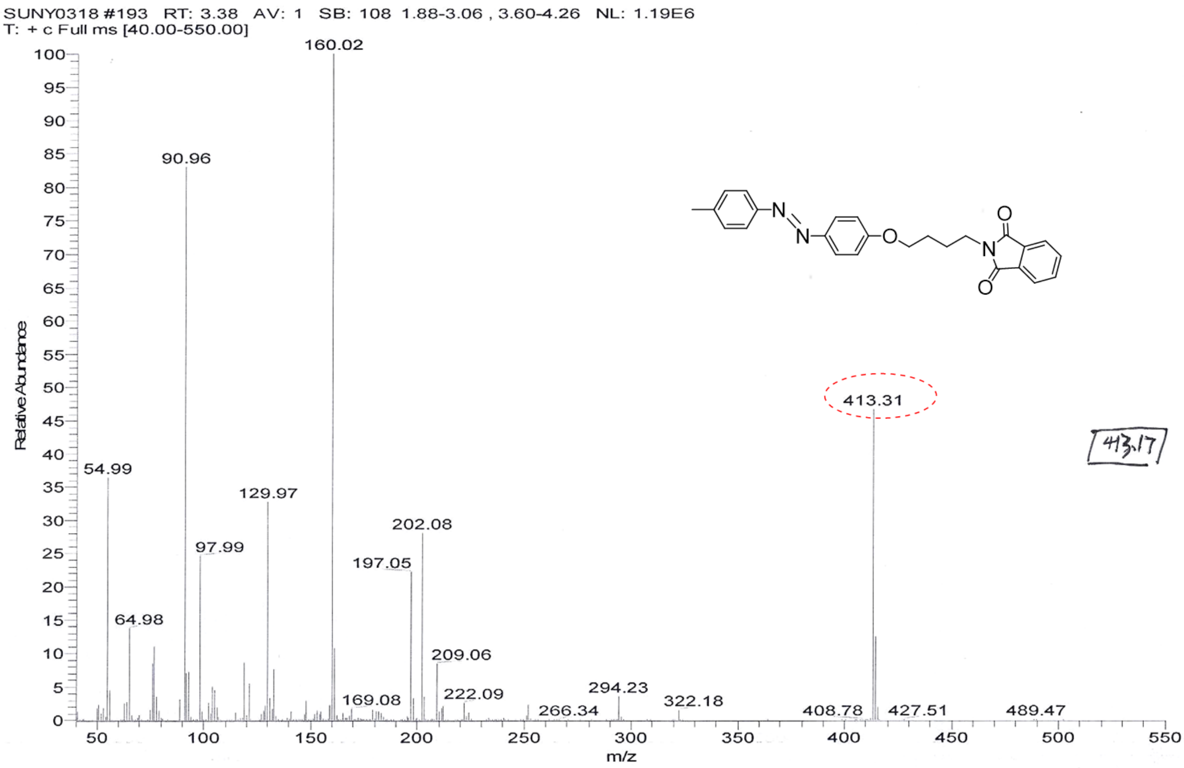

Supplementary Figure 33. Mass spectrum of compound **G3**

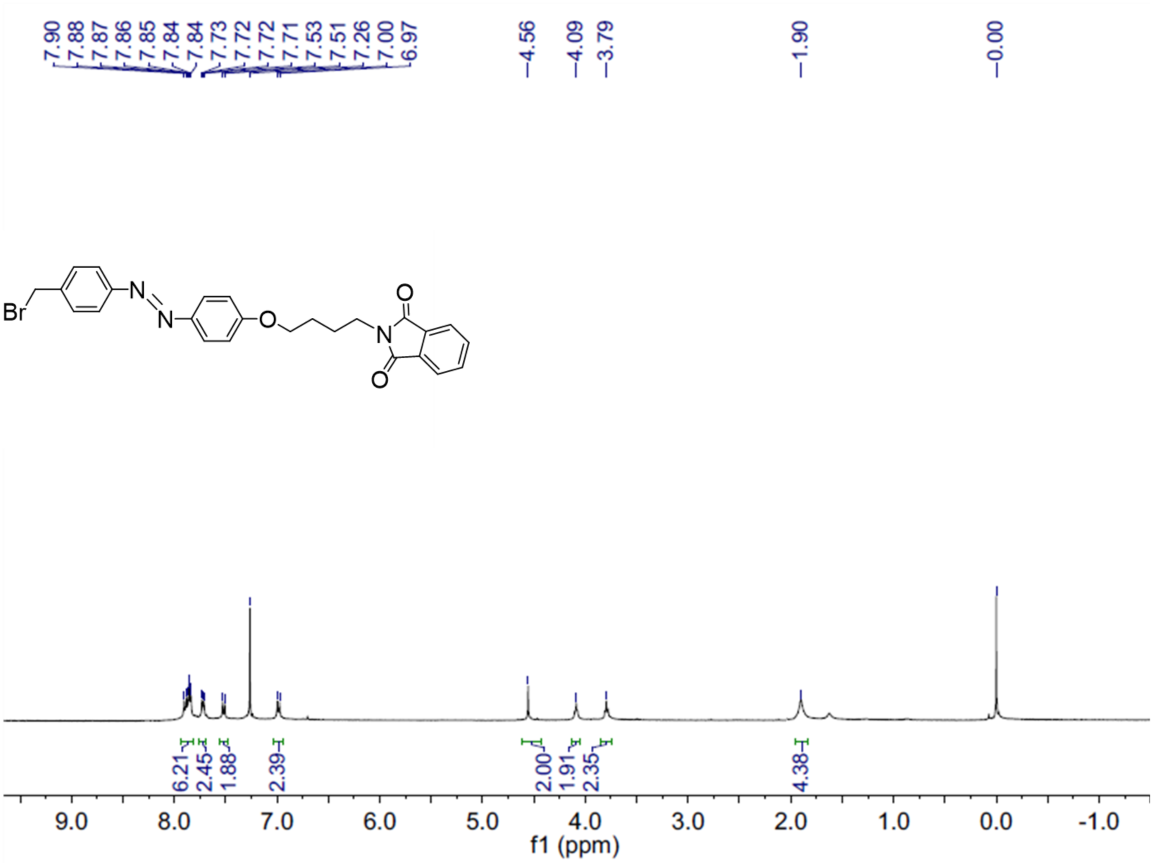

Supplementary Figure 34. ^{1}H NMR spectrum (400 MHz) of **G2** in $CDCl_3$

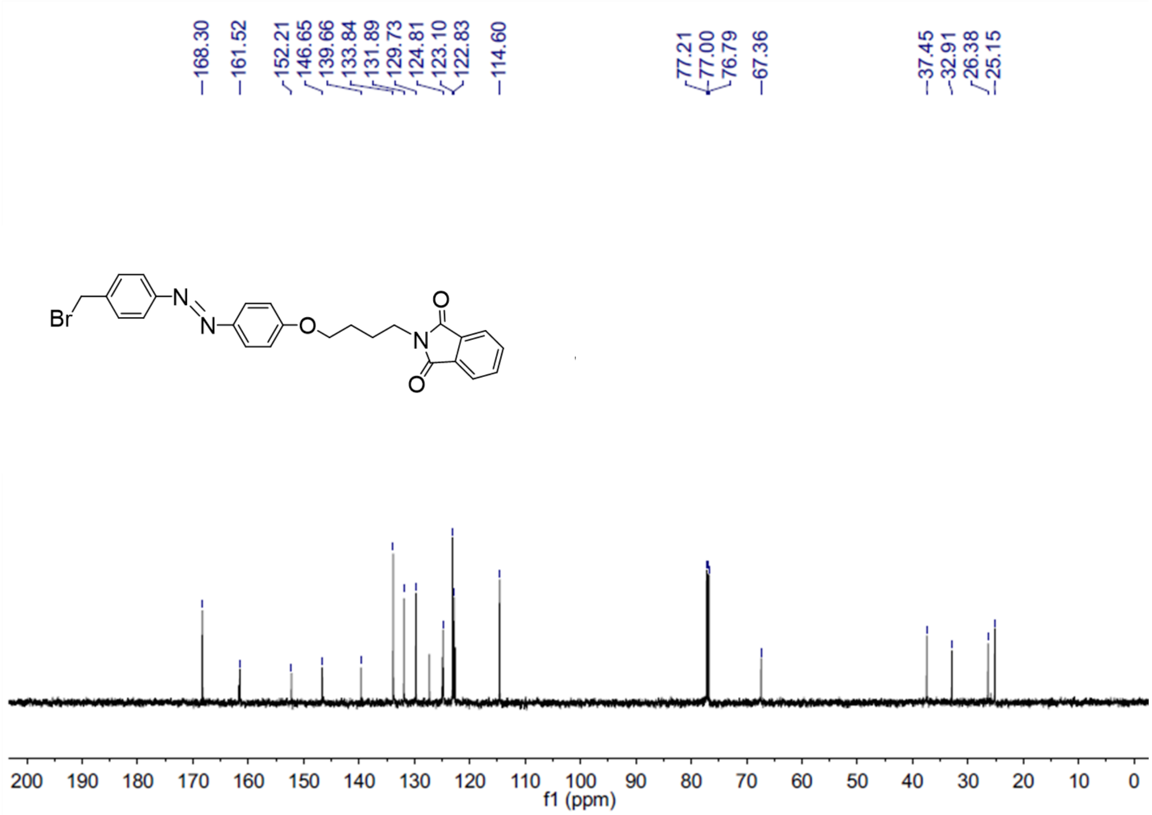

Supplementary Figure 35. ^{13}C NMR spectrum (100 MHz) of **G2** in $CDCl_3$

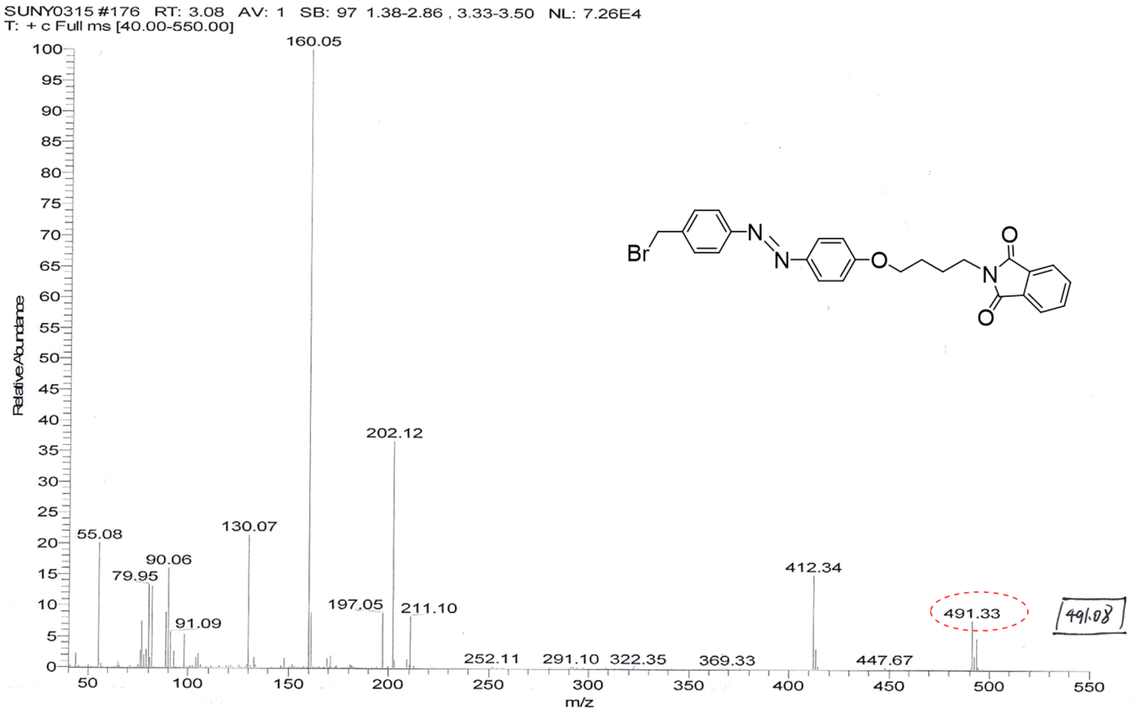

Supplementary Figure 36. Mass spectrum of compound **G2**

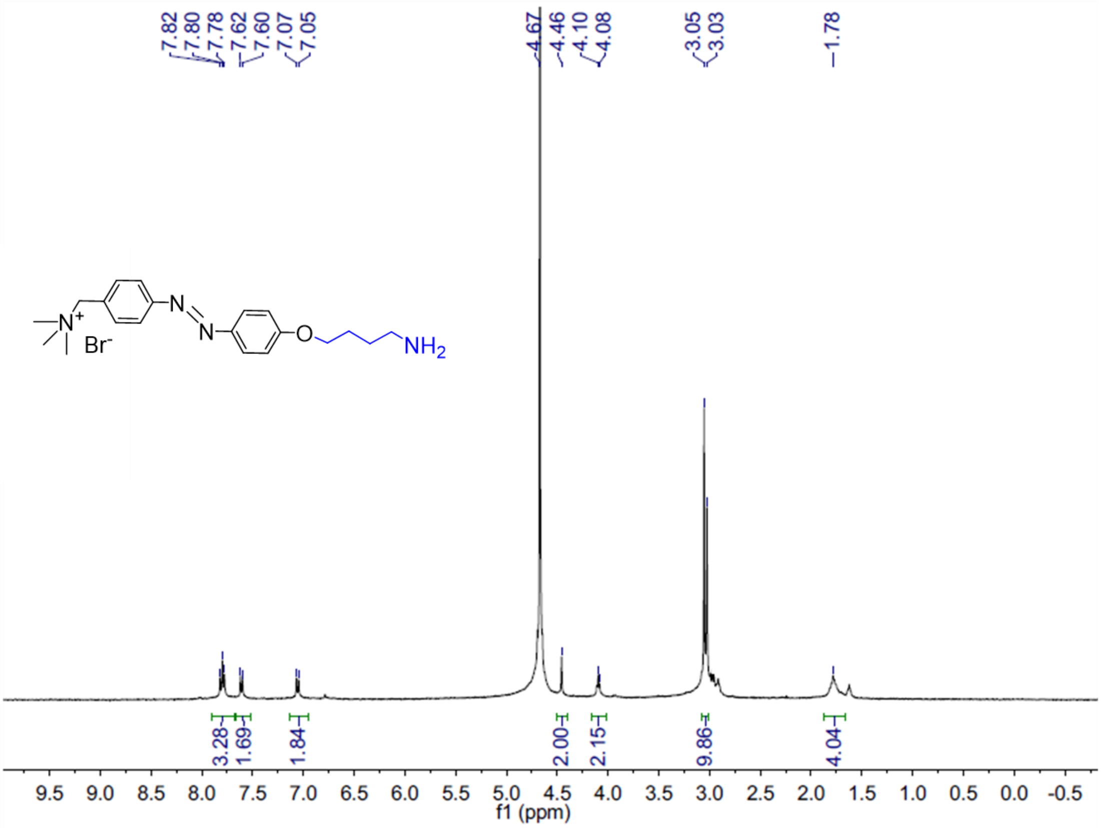

Supplementary Figure 37. ^{1}H NMR spectrum (400 MHz) of **G1** in D_2O

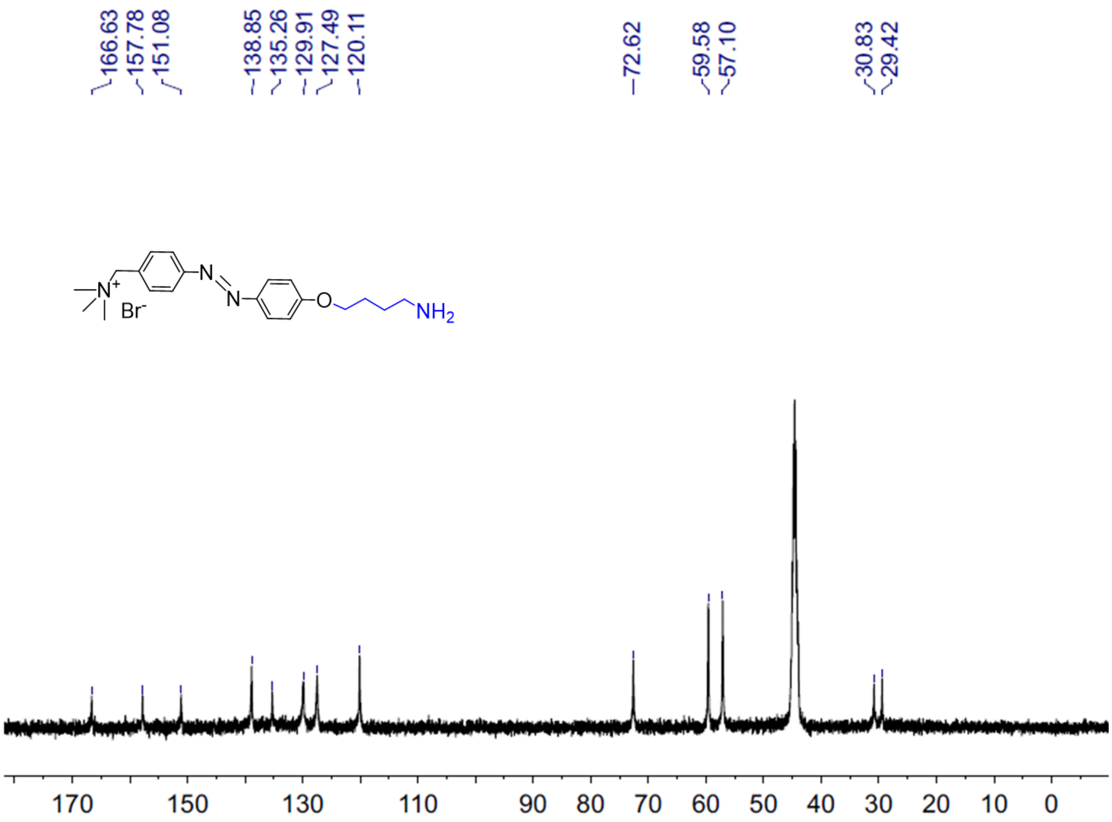

Supplementary Figure 38. ^{13}C NMR spectrum (100 MHz) of **G1** in DMSO

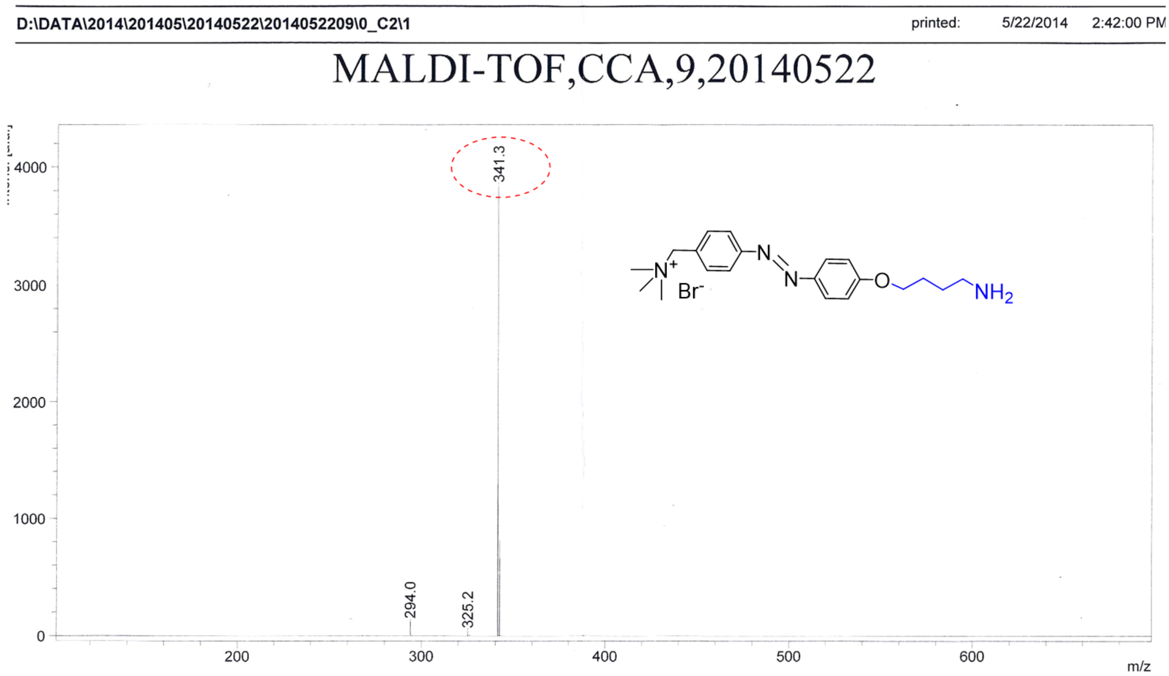

Supplementary Figure 39. Mass spectrum of compound **G1**

Supplementary Methods

Materials. Poly (ethylene terephthalate) (PET, 12 μm thick) membranes were irradiated with single heavy ion (Au) of energy 11.4 MeV/nucleon at UNILAC linear accelerator (GSI, Darmatadt, Germany). 1-Ethyl-3-(3-dimethyllaminopropyl) carbodiimide hydrochloride (EDC·HCl, ≥98.5%), N-hydroxysulfosuccinimide (NHS, ≥98.0%), sodium hydroxide (NaOH), hydrochloric acid (HCl), formic acid (HCOOH), potassium chloride (KCl) were purchased from Sinopharm Chemical Reagent Shanghai Co., Ltd. (SCRC, China). All chemical reagents were all used as received, electrolyte solution were prepared in MilliQ water (18.2 MΩ). Current-voltage curves were measured by a Keithley 6487 picoammeter (Keithley Instruments, Cleveland, OH). Confocal fluorescent images were acquired using a Zeiss confocal laser scanning unit mounted on a LSM710 fixed-stage upright microscope. For UV light irradiation, a 300 W xenon lamp was used with a 365 nm filter. The intensity was measured with an optical power/energy meter (Model 842-PE). For this work, a custom-built, photoelectro-chemical cell was adopted, which can be irradiated from both sidewalls. Scanning electron microscopy (SEM) investigations were carried out on a JEOL 6390LV instrument.

Synthetic and characterization of L-AP6 and AZO.

Reagents were commercially available and used as received. Solvents were either employed as purchased or dried according to procedures described in the literature. ^{1}H and ^{13}C NMR spectra were recorded on a Mercury-Plus spectrometer (400 MHz). MALDI-TOF-TOF were recorded on a Synapt G2 HDMS system (Waters,USA). Elemental analyses were performed on a Perkin-Elmer 240 C analyzer.

Synthesis of compound H4 : Compound H5 was synthesized according to the literature.[S1] Sodium azide (390 mg, 6.0 mmol) was added to the solution of compound H5 (500 mg, 0.25 mmol) in anhydrous DMF (10 ml). After stirring at 100 °C for 12 h under nitrogen atmosphere, the mixture was cooled to room temperature and poured into water (80 mL). The precipitate was collected by filtration, and washed with water to yield compound H4 as white solid (367 mg, 95%). ^{1}H NMR (400 MHz, $CDCl_3$): δ 6.74 (s, 12H), 3.97 (s, 24H), 3.87 (s, 12H), 3.46 (s, 24H) ppm. ^{13}C NMR (100 MHz, $CDCl_3$): δ 149.75, 128.75, 115.51, 67.35, 50.86, 29.51 ppm. MALDI-TOF-MS: Calcd for $C_{66}H_{72}O_{12}N_{36}$: 1560.61. Found: 1583.60, $[M+Na]^+$. Anal. Calcd for $C_{66}H_{72}O_{12}N_{36}$: C, 50.77; H, 4.65; N, 32.29; found: C, 50.80; H, 4.60; N, 32.32. ***On account of the**

distinct possibility of generating diazidomethane in the extraction step, this reaction should not be performed on a large scale.

Synthesis of compound H3: A suspension of compound **H4** (310 mg, 0.2 mmoL) and Pd/C (10%, 50 mg) in methanol was stirred at 50 °C under hydrogen atmosphere (10 atm) for 24 h. The resulting mixture was filtered, and the filtrate was concentrated under reduced pressure. The residue was purified by crystallization from chloroform to produce compound **H3** as white solid (240 mg, 96%). ^{1}H NMR (400 MHz, CH_3OH-*d4*): δ 6.72 (s, 12 H), 3.89 (s, 12 H), 3.83 (d, *J* = 4.0 Hz, 12 H), 2.90 (s, 12 H) ppm. ^{13}C NMR (100M Hz, CH_3OH-*d4*): δ 148.74, 127.95, 114.41, 66.92, 38.64, 27.99 ppm. MALDI-TOF-MS: Calcd for $C_{66}H_{96}O_{12}N_{12}$: 1226. 95. Found: 1249.97, $[M+Na]^+$. Anal. Calcd for $C_{66}H_{96}O_{12}N_{12}$: C, 63.44; H, 7.74; N, 13.45; found: C, 63.39, H, 7.77; N, 13.49.

Synthesis of compound H2: To a suspension of compound H3 (125 mg, 0.1 mmol) and 2-(1H-Benzotriazole-1-yl)-1,1,3,3-tetramethyluronium hexafluorophosphate (HBTU) (547 mg, 1.44 mmol) in dry CH_2Cl_2 (20 ml) N-(tert-Butoxycarbonyl)-L-alanine (228 mg, 1.44 mmol) and NEt_3 (0.2 mL, 1.44 mmol) are added. The mixture is stirred at room temperature 4 h, then HCl (1.0 M, 10 mL) is added and the organic layer is washed with H_2O, aqueous solution of $NaHCO_3$ (5%, 10 mL), H_2O (10 mL) and finally concentrated. The pure product H2 is obtained by flash chromatography on silica gel (CH_2Cl_2 / CH_3OH = 5: 1) (198 mg, yield: 65 %). ^{1}H NMR (400 MHz, $CDCl_3$): δ 7.72 (d, *J* = 12.0, 12H), 6.72 (br. s, 12H), 5.94 (br. s, 12H), 4.34 (br. s, 12H), 3.93 (m, 48H), 1.43 (br. s, 108H) ppm. ^{13}C NMR (100M Hz, $CDCl_3$): δ 173.67, 155.43, 150.06, 128.21, 114.09, 79.56, 67.05, 49.91, 38.98, 28.15, 28.03, 18.38 ppm. MALDI-TOF-MS: Calcd for $C_{162}H_{252}O_{48}N_{24}$: 3321.79. Found: 3324.80, $[M+Na]^+$. Anal. Calcd for $C_{162}H_{252}O_{48}N_{24}$: C, 58.89; H, 7.69; N, 10.17; found: C, 58.85; H, 7.70; N, 10.14.

Synthesis of compound H1: Compound **H2** (100 mg, 0.019 mmol) was dissolved in TFA (2 mL). After 5 h, the mixture was evaporated and dried under vacuum to afford Compound H1 as its trifluoroacetate salt. The above material was dissolved in the water and neutralised by the addition of 0.5 M NaOH aq. until pH = 7. Then removed the solvent and recrystallized with methanol to yield the white compound (41 mg, 92%). ^{1}H NMR (400 MHz, D_2O): δ 6.59 (s, 12H), 3.68 (s, 24 H), 3.65 (s, 12H), 3.33 (s, 12H), 3.29 (d, 24H), 1.02 ppm (d, *J* = 4.0, 36H) ppm; ^{13}C NMR (100 MHz, CH_3OH-*d4*): δ 160.50, 160.17, 149.20, 126.83, 116.99, 114.06, 113.80, 65.65, 38.04, 15.15 ppm.

MALDI-TOF-MS: Calcd for $C_{102}H_{150}O_{24}N_{24}$: 2101.16; found: 2124.23, $[M+Na]^+$. Anal. Calcd for $C_{102}H_{150}O_{24}N_{24}$: C, 58.44; H, 7.21, N, 16.03; found: C, 58.55; H, 7.26; N, 16.00.

Synthesis of compound (G3): Compound **G4** was synthesized according to the literature.[S2] Phthalimide (0.184 g, 1.25 mmol) and K_2CO_3 (0.345 g, 2.5 mmol) was added to a solution of G4 (266 mg, 1.00 mmol) in dry N, N-dimethylformamide (30 mL). The reaction mixture was stirred at ambient temperature for 12h under the protection of nitrogen atmosphere. Then the DMF solvent was removed under vacuum to give buff solid. The residue was dissolved with chloroform. The organic layer was washed with H_2O. The organic layer was dried over Na_2SO_4. After the solvent was evaporated, the residue was purified by column chromatography (silica gel, hexane–dichloromethane, 1 : 1) to give **G3** as yellow product (363 mg, yield: 88%). ^{1}H NMR (400 MHz, $CDCl_3$): δ 7.87 (s, 4H), 7.79 (s, 2H), 7.73 (s, 2H), 7.72 (s, 2H), 6.99 (d, J = 12.0 Hz, 2H), 4.08 (s, 2H), 3.80 (s, 2H), 2.43 (s, 2H), 1.90 (s, 4H). ^{13}C NMR (100 MHz, $CDCl_3$): δ 168.22, 161.10, 150.61, 146.74, 140.58, 133.76, 131.87, 129.52, 124.40, 123.02, 122.38, 114.48, 67.26, 37.42, 26.38, 25.14, 21.32. MALDI-TOF-MS: caclulated for $C_{25}H_{23}N_3O_3$: 413.17, found 413.31. Anal. Calcd for $C_{25}H_{23}N_3O_3$: C, 72.62; H, 5.61; N, 10.16; found: C, 72.66; H, 5.57; N, 10.17.

Synthesis of compound (G2): A mixture of **G3** (413mg, 1 mmol), N-bromosuccinimide (0.25 g, 1.4 mmol) and benzoyl peroxide (10 mg, 0.042 mmol) in CCl_4 (20 mL) was heated at reflux for 12 h. The mixture was cooled to room temperature and washed with water (2x30 mL), dried (Na_2SO_4) and concentrated in vacuo. The residue was purified by column chromatography (silica gel, hexane-dichloromethane, 1 : 1) to give compound G2 (360 mg, yield: 73%). ^{1}H NMR (400 MHz, $CDCl_3$): δ 7.90 (m, J = 8.0 Hz, 6H), 7.85 (d, J = 4.0 Hz, 2H), 7.73 (d, J = 4.0 Hz, 2H), 7.00 (d, J = 12.0 Hz, 2H), 4.56 (s, 2H), 4.09 (s, 2H), 3.79 (s, 2H), 1.90 (s, 4H). ^{13}C NMR (100 MHz, $CDCl_3$): δ 168.30, 161.52, 152.21, 146.65, 139.66, 133.84, 131.89, 129.73, 124.81, 123.10, 122.83, 114.60, 67.36, 37.45, 32.91, 26.38, 25.15. MALDI-TOF-MS: Calcd for $C_{25}H_{22}N_3O_3Br$: 491.33; found 491.33. Anal. Calcd for $C_{25}H_{22}N_3O_3Br$: C, 60.98; H, 4.50; N, 8.53; found: C, 60.99; H, 4.53; N, 8.49.

Synthesis of compound (G1): A solution of **G2** (493 mg, 1 mmol) in ethanol (50.0 mL) and trimethylamine (30% in ethanol, 10.0 mL) was allowed to react at 80 °C for 24 h. After that, hydrazine hydrate was added to the mixture, to futher reaction for 12 h.

The solution was concentrated under reduced pressure. The residue was diluted with water (20.0 mL) and washed with dichloromethane. Then, removed water in vacuo to give a organe solid. (145 mg, 41%).^{1}H NMR (400 MHz, $CDCl_3$): δ 7.82 (m, *J* = 8.0 Hz, 4H), 7.62 (d, *J* = 8.0 Hz, 2H), 7.07 (d, *J* = 8.0 Hz, 2H), 4.46 (s, 2H), 4.10 (d, *J* = 8.0 Hz, 2H), 3.05 (s, 9H), 1.78 (s, 4H). ^{13}C NMR (100 MHz, DMSO): δ 166.63, 157.78, 151.08, 138.85, 135.26, 129.91, 127.49, 120.11, 72.62, 59.58, 57.10, 30.83, 29.42. MALDI-TOF-MS: Calcd for $C_{20}H_{29}N_4OBr$: 420.15. Found: 341.3 [M-Br^-]. Anal. Calcd for $C_{20}H_{29}N_4OBr$: C, 57.01; H, 6.94; N, 13.30; found: C, 56.97; H, 6.98; N, 13.30.

The interaction between AZO and L-AP6

To determine the stoichiometry and association constant (K_a) between L-AP6 and AZO. ^{1}H NMR titrations were done with solutions which had a constant concentration of L-AP6 (4 mM) and varying concentrations of AZO. By a mole ratio plot, a 1:1 stoichiometry was obtained, which indicated that L-AP6 and AZO formed a 1:1 complex. Using the nonlinear curve-fitting method, the association constant was obtained for each host-guest combination from the following equation : [S3]

$$\Delta\delta = (\Delta\delta_\infty/[H]_0)\ (0.5[G]_0 + 0.5([H]_0 + 1/K_a) - (0.5\ ([G]_0{}^2 + (2[G]_0(1/K_a - [H]_0)) + (1/K_a + [H]_0)^2)\ ^{0.5}))$$

Where is the chemical shift change of H_a of amide in L-AP6 at $[G]_0$, is the chemical shift change of H_a when the host is completely complexed, $[H]_0$ is the fixed initial concentration of the host L-AP6, and $[G]_0$ is the varying concentrations of guest AZO.

Fabrication of single conical nanochannel

The single conical nanochannel was prepared in a PET polymer film using the well-known ion track etching technique. Before etching process, each side of the PET membranes were exposed in UV light (365 nm) for 1 h. In order to obtain the conical nanochannel, etching was performed only from one side, the other side of the cell contains a solution that is able to neutralize the etchant as soon as the pore opens, thus slowing down the further etching process. The PET membrane was embedded between the two chambers of a conductivity cell at 30 °C, one chamber was filled with etching solution (9 M NaOH), the other chamber was filled with stopping solution (1 M KCl + 1 M HCOOH). Then a voltage of 1 V was applied across the membrane. The etching process was stopped at a desired current value corresponding to a certain tip diameter. The membrane was immerged in MilliQ water (18.2 MΩ) to remove residual salts. The diameter of large opening of conical nanochannel which was called base

(D) was determined by scanning electron microscopy (SEM). The diameter of the small opening which was called tip (d_{tip}) was estimated by the following relation:

$$d_{tip} = \frac{4LI}{\pi k(c)UD}$$

L is the length of the pore, which could be approximated to the thickness of the membrane after chemical etching; I is the measured ion current; U is the applied voltage; d_{tip} and D is the tip diameter and the base diameter respectively; k(c) is the specific conductivity of the electrolyte. For 1 M KCl solution at 25 °C, k(c) is 0.11173 Ω^{-1} cm^{-1}. In this work, the base diameter is about 580 nm and the tip diameter is about 18 nm, which was further confirmed by SEM.

SEM Characterization

The diameter of the base was estimated from the multitrack membrane by field-emission scanning electron microscopy (FESEM) which was etched under the same conditions as the single-channel sample. In this work, before modification the base diameter was about 580 nm and tip diameter was estimated by the above relation, tip was about 18 nm.

The modification process of chiral nanochannel

As a result of chemical etching, carboxyl groups are generated on the nanochannel surface. These can be activated with EDC/NHS, forming an amine-reactive ester intermediate. Then these reactive esters were further condensed with AZO through the formation of covalent bonds. In this paper NHS ester was formed by soaking PET film in an aqueous solution of 30 mg EDC and 6 mg NHS for 1 hour. After that washing this film with distilled water and treated it with 1 mM AZO solution overnight. Then, the L-AP6 were attached to the AZO-channel by self-assembling. Finally, the modified-film was washed three times with distilled water.

Contact angles measurement

Contact angles were measured using an OCA20 (DataPhysics, Germany) contact angle system at ambient temperature and saturated humidity. The original PET membrane for contact angle measurement was treated with NaOH (9 M) at 38 °C for 50 min. And then the sample was removed from the etching solution and treated with the stopping solution (1 M HCOOH) for 20 min. After that, the sample was treated with distilled water overnight. The modification process on the PET film is same to the

modification process in the inner wall of the nanochannel. Before the contact angle test, the sample was blown dry with N_2. In each measurement, an about 1μL droplet of water was dispensed onto the surface of PET membrane. The average contact angel value was obtained at five different positions of the same membrane. As shown in Figure S5, the change of the wettability of the surface means the change of the chemical composition, to some extent, which indicated the successful modification of the AZO and L-AP6.

XPS experiment

X-ray photoelectron spectra (XPS) data were obtained with an ESCALab220i-XL electron spectrometer from VG Scientific using 300 W Al K_α radiation. In this work, to further prove the L-AP6 and AZO modified successfully by measuring the nitrogen element. L-AP6 was labelled by Dansyl chloride (DNS). All peaks were referenced to C1s (CHx) at 284.8eV in the deconvoluted high resolution C1s spectra. The nitrogen element existed in the PET film indicates that AZO was modified on the surface of the film successfully. Furthermore, the sulfur element show that the film was decorated with L-AP6-DNS.

The K values (binding constants) in the nanochannels

In previous literature, it was discovered that the Langmuir model provided a perfect fit to the experimental data for nanochannels.[S4] In our work, furthermore, the variation of the rectification ratio ($R_{-/+}$) with increasing concentration of saccharides, was similar to the trend of the Langmuir absorption isotherm. Consequently, we speculated that it would be possible to describe the binding of saccharides enantiomers on the internal surface of the L-AP6-channel using the Langmuir model.

The electroosmotic flow (EOF) experiments

We conducted a series of electroosmotic flow (EOF) experiments, which is an electrokinetic phenomenon that occurs when an ionic current is passed through nanochannel that contains excess surface charge. EOF was driven through the membrane by using a Pt electrode in each half-cell solution to pass a voltage of 6V through the channels. Because PET surfaces have excess negative charge, EOF is in the direction of cation migration. For this reason, the cathode was in the permeate solution for all EOF experiments. The phenol is used as an electrically neutral probe to measure the EOF rate from the feed to the permeate. This was accomplished by

periodically measuring the fluorescence emission of the phenol in the permeate solution and making plots of moles of phenol transport vs time. We may caculate the V_{eof}, and then surface charge density values can be estimated from the Gouy-Chapman equation. As shown in Supplementary Fig. 17, surface charge density vs Log (concentration of Glu). To some extent, it can explain the reason for the chiral selectivity in nanochannel, which may be attributed to the different surface charge densities on the channel walls generated by the binding of D-Glu/L-Glu.

The surface pK_a [S5, S6]

Measuring the current at the -2 V for the couple as a function of pH allows the determination of the pK_a of the surface. The equation commonly used in the buffer region of the acid–base equilibrium is the Henderson–Hasselbach equation:

$$\mathrm{pKa} = \mathrm{pH} - \log(\frac{[\mathrm{A}^-]}{[\mathrm{HA}]}) \qquad \text{(Supplementary Equation 1)}$$

The method for determining surface pK_a uses the following argument: if the total current through the electrode is assumed to be composed of two independent parts, one through the dissociated Channel [A−] and the other through the non-dissociated Channel [HA], then the current can be described by

$$i = i_{\mathrm{A}^-}[\mathrm{A}^-] + i_{\mathrm{HA}}[\mathrm{HA}] \quad \text{(Supplementary Equation 2)}$$

where i_{A^-} and i_{HA} are currents of the probe on the channel fabricated by [A−] and [HA] structures, respectively. In this case, [HA] refers to surface concentration, not solution concentration. By setting the channel coverage to 1, the coverage of the surface components is [A−] + [HA] = 1. Using this expression, plus eqs 1 and 2, the following equation can be obtained,

$$\mathrm{pKa} = \mathrm{pH} - \log(\frac{i_{\mathrm{HA}} - i_{\mathrm{A}^-}}{i - i_{A^-}} - 1) \quad \text{(Supplementary Equation 3)}$$

where i_{A^-} and i_{HA} can be determined by the average values of the high pH (> 6) and low pH (< 4), respectively. Hence, a plot of peak current i versus pH can elucidate the pK_a of a surface.

The current at -2 V can be determined and plotted against pH as shown in Supplementary Fig. S18. This plot looks similar to the equivalence part of a titration curve, and using eq 3, the pK_a can be elucidated. Hence, the surface pK_a at the neutral condition before adding the D-/L-Glu is approximately 6.08. After binding with glucose enantiomers, the surface pK_a at the neutral condition is approximately 6.71 for binding D-Glu and is approximately 6.14 for binding L-Glu.

Supplementary References

1. Jie, K. et al. *Chem. Soc. Rev.,* 44, 3568–3587(2015).
2. Ito, M. et al. *Chem*. 15, 478–483 (2005).
3. Li, C. et al. *Chem. Commun*. 46, 9016–9018 (2010).
4. Nie, G. R. et al. *Chem. Sci*. 6, 5859–5865 (2015).
5. Munakata. H. et al. *Chem. Commun*. 1338–1339 (2001).
6. Hale, P. S. et al. *J. Chem. Educ.*, 82, 779–781 (2005)
